# WAFER trial: a study protocol for a feasibility randomised controlled trial comparing wide-awake local anaesthesia no tourniquet (WALANT) to general and regional anaesthesia with tourniquet for flexor tendon repair

**DOI:** 10.1136/bmjopen-2023-075440

**Published:** 2023-08-28

**Authors:** Muholan Kanapathy, Ryan Faderani, Juliette Bray, Hakim-Moulay Dehbi, Monica Panca, Cecilia Vindrola-Padros, Anjana Prasad, Nicola Burr, Norman R Williams, Yazan Al-Ajam, Waseem Bhat, Jason Wong, Afshin Mosahebi, Dariush Nikkhah

**Affiliations:** 1 Division of Surgery and Interventional Science, University College London, London, UK; 2 Department of Plastic and Reconstructive Surgery, Royal Free NHS Foundation Trust Hospital, London, UK; 3 Faculty of Medical Sciences, University College London, London, UK; 4 University College London Institute of Clinical Trials and Methodology, London, UK; 5 Comprehensive Clinical Trials Unit, University College London, London, UK; 6 Department of Targeted Intervention, University College London, London, UK; 7 Department of Anaesthesia, Royal Free NHS Foundation Trust Hospital, London, UK; 8 Department of Plastic and Reconstructive Surgery, Leeds Teaching Hospitals NHS Trust, Leeds, UK; 9 Blond McIndoe Laboratories, Division of Cell Matrix Biology and Regenerative Medicine, Faculty of Biology Medicine and Health, The University of Manchester, Manchester, UK

**Keywords:** ANAESTHETICS, Anaesthesia in orthopaedics, Hand & wrist, PLASTIC & RECONSTRUCTIVE SURGERY, Plastic & reconstructive surgery

## Abstract

**Introduction:**

Flexor tendons are traditionally repaired under either general anaesthesia (GA) or regional anaesthesia (RA), allowing for the use of an arm tourniquet to minimise blood loss and establish a bloodless surgical field. However, the use of tourniquets exposes the patient to certain risks, including skin, muscle and nerve injuries. A recent advancement in anaesthesia delivery involves the use of a wide-awake approach where no sedation nor tourniquets are used (wide-awake local anaesthesia no tourniquet (WALANT)). WALANT uses local anaesthetic with epinephrine to provide pain relief and vasoconstriction, reducing operative bleeding. Several studies revealed potential benefits for WALANT compared with GA or RA. However, there remains a paucity of high-quality evidence to support the use of WALANT. As a result of this uncertainty, the clinical practice varies considerably. We aim to evaluate the feasibility of WALANT as an alternative to GA and RA in patients undergoing surgical repair of flexor tendon injuries. This involves addressing factors such as clinician and patient support for a trial, clinical equipoise, trial recruitment and dropout and the most relevant outcomes measures for a future definitive trial.

**Methods and analysis:**

WAFER is a multicentre, single-blinded, parallel group, randomised controlled trial (RCT) to assess the feasibility of WALANT versus RA and GA. The target population is patients with acute traumatic flexor tendon injuries, across 3 major hand surgery units in England involving a total of 60 participants. Outcome assessors will be blinded. The primary outcome will be the ability to recruit patients into the trial, while secondary outcomes include difference in functional outcome, patient-reported outcome measures, health-related quality of life, cost-effectiveness and complication rates.

**Ethics and dissemination:**

Ethical approval was obtained from the London—City and East Research Ethics Committee (22/PR/1197). Findings will be disseminated through peer-reviewed publication, conferences, patient information websites and social media networks.

**Trial registration number:**

ISRCTN identifier: 15052559.

**STRENGTHS AND LIMITATIONS OF THIS STUDY**

- This study will use mixed methods which include quantitative and qualitative measures to assess the feasibility of conducting a randomised controlled trial on performing flexor tendon repair under wide-awake local anaesthesia no tourniquet compared with general or regional anaesthesia with tourniquet.
- The inclusion of comprehensive outcome measures in the study will offer valuable insights into both the objective and patient-reported impact of flexor tendon injury.
- This study includes several major hand surgery units from different regions of the country to be inclusive and to identify differences and challenges faced at different units that are recruiting into the same trial to facilitate future study design.
- This study was designed and run by plastic surgical trainees alongside consultant plastic surgeons and surgical trials unit which, emphasises the important role that surgical trainees play in surgical research.
- One limitation of this study is its feasibility nature, which means that the sample size is not sufficient to detect differences between groups with a high degree of confidence.

## Introduction

Hand injuries account for up to 20% of all presentations to emergency departments, equating to 4.6 million patients annually in the UK alone and resulting in an average of 8 weeks of lost workdays.^1 2^ In patients with hand injuries, tendon lacerations are common and are reported in 54.8% of patients with a shallow laceration and 92.5% with deeper injury.^3^ Patients with complete tendon laceration will require surgical repair, which is typically performed in the operating room, using an arm tourniquet to minimise blood loss and provide a clear surgical field.^4^ The use of a tourniquet is associated with significant discomfort and is not well tolerated by patients without general anaesthesia (GA) or regional anaesthesia (RA).^5^

A recent advancement in the method of anaesthesia delivery in hand surgery involves the introduction of wide-awake local anaesthesia no tourniquet (WALANT).^6–8^ WALANT involves administering local anaesthesia (LA) with epinephrine for anaesthetic and vasoconstriction effect, respectively.^8^ The vasoconstrictive effect of epinephrine achieves reduced bleeding at the site of surgery, hence acting as a chemical tourniquet and eliminating the need for a mechanical arm tourniquet. Removing the need for tourniquet and therefore GA/RA has several potential advantages for patients. First, patients can make immediate postoperative recovery, ultimately shortening the patient stay in hospital. GA is associated with side effects such as nausea and vomiting along with serious cardiac and respiratory risks especially among patients undergoing emergency surgery, elderly, smokers and overweight patients.^9^ RA avoids these risks but exposes the patient to the risk of permanent or temporary nerve damage (1 in 10) and failure of providing adequate pain relief resulting in conversion to GA (3 in 100).^10 11^ Tourniquet use has also been linked to increased postoperative pain and side effects such as muscle, nerve and skin injury and post-tourniquet thrombosis.^5 12^

While WALANT requires multiple injections that may cause temporary discomfort, it avoids many of the significant issues associated with GA and RA. Furthermore, patients who undergo WALANT have been reported to have a decreased need for post-operative pain relief.^13^ Additionally, WALANT allows the surgeon to intraoperatively evaluate the tendon repair, while the patient actively moves their fingers, allowing adjustments to be made before skin closure. This has been shown to improve surgical outcomes and reduce the need for revision surgery.^14 15^ Patients can also receive education during the procedure to aid their rehabilitation.^7^ There are also potential economic and environmental benefits to using WALANT, as it does not require an anaesthetist, requires fewer clinical staff (anaesthetist, anaesthetic assistant, recovery nurse) and produces fewer anaesthetic waste gases.^16 17^

### Rationale

In recent years, WALANT has gained significant popularity as an alternative to GA or RA with tourniquet for flexor tendon repairs.^18^ While studies have suggested that it may have similar safety and potential benefits in comparison to GA or RA with a tourniquet, it should be noted that these studies are either retrospective in nature or lack sufficient statistical power to make definitive conclusions.^19–22^ Furthermore, it is important to determine the outcomes that are important to be measured, evaluate equipoise among clinicians to recruit to a study comparing WALANT against GA/RA and acceptability of the trial to patients and clinicians before a definitive trial can be carried out. In addition, because of the uncertainty about the effectiveness and cost-effectiveness of WALANT compared with standard of care and the variation is clinical practice, a definitive randomised controlled trial (RCT) on the effectiveness and cost-effectiveness of WALANT is necessary. A feasibility study is hence necessary to help with the design of the definitive RCT. The WAFER trial therefore aims to evaluate the feasibility of WALANT as an alternative to GA or RA in patients undergoing surgical repair of traumatic laceration of flexor tendons of the hand.

## Methods and analysis

### Trial design

The WAFER trial is a multicentre, assessor-blinded, feasibility RCT with two parallel groups (WALANT vs GA/RA) conducted in three sites within the UK. Eligible patients will be randomised to WALANT or GA/RA for flexor tendon repair using a computerised randomisation method. This protocol is reported in accordance with Standard Protocol Items: Recommendations for Interventional Trials 2013 guidelines.^23^

### Patient and public involvement

Patient representatives who have had flexor tendon injury were involved in the design of this trial by providing feedback via online surveys and focus group meetings. A total of 27 patients were involved in the online survey and another 12 patients were involved in focus group meetings to identify priorities and outcomes of a flexor tendon study. Two patient representatives have been involved in designing and revising the trial to ensure that patient interests are central to the study design and that the trial is written clearly avoiding excessive or unnecessary medical jargon.

### Study setting

Participants will be recruited at the Royal Free Hospital, Manchester University National Health Service (NHS) Foundation Trust, and Leeds Teaching Hospital NHS Trust. This trial is open to recruiting additional sites through National Institute for Health Research Clinical Research Network.

### Eligibility criteria

Patients presenting to the accident and emergency unit or outpatient clinics with acute traumatic flexor tendon ruptures will be reviewed by a hand surgeon and assessed. Normal clinical practice is that all patients will be assessed clinically and if the flexor tendon is lacerated, it will be scheduled for repair under GA/RA or WALANT in the operating theatre. On scheduling the patient for surgery, patients will be flagged to the research team. At this time, patients will be screened and where appropriate be offered a patient information sheet for inclusion into the trial. This process will include explanation of the aims, methods of anaesthesia and surgery, anticipated benefits and potential hazards of the study. Patients will be given sufficient time (24 hours or more if needed) to consider whether they wish to participate. Once the patients are ready for surgery, following review by the study team, patients will be assessed clinically to confirm ‘inclusion and exclusion criteria’ are met and an informed consent form will be completed, following which patients will be randomised to either of the treatment arms and enrolled into the study. Patient related data will be recorded in a patient assessment form.

### Inclusion criteria

1. Adults≥18 years old.
2. Clinical diagnosis of acute flexor tendon injury within 2 weeks of injury (≤2 weeks from injury to date of surgery).
3. Complete laceration (100% transection) of flexor digitorum profundus, and/or flexor digitorum superficialis, and/or flexor pollicis longus of the hand.
4. Single digit or two digits injury involving flexor zones 1–3.
5. The patient understands and is willing to participate and can comply with the follow-up regime.

### Exclusion criteria

1. Tendon not amendable to primary repair (gross wound contamination, segmental tendon loss, associated fractures or mangled hand injuries).
2. Secondary tendon repair or reconstruction.
3. History of allergy to LA.
4. Refusal to have LA or deemed non-cooperative to be performed without sedation.
5. Pre-existing deformity of finger or hand.
6. High risk for GA or not fit for surgery (American Society of Anesthesiologists (ASA)≥4).

### Interventions

#### Wide-awake local anaesthesia no tourniquet

Patients will have the procedure in an operating theatre under WALANT without an arm tourniquet. WALANT uses a mixture of LA with epinephrine to provide anaesthesia and haemostasis. A mixture of 1% xylocaine with epinephrine 1:200 000 will be injected into the digit or hand requiring surgery. This mixture is routinely used in clinical practice.^24 25^ The mixture is used in a ‘field block’ technique where 2 mL is injected into the subcutaneous fat in the middle of the base of the proximal and middle phalanges between both digital nerves, and 1 mL is injected into the distal phalanx. In addition, 10–15 mL LA will be injected in the palm where dissection will be performed.^26^

Following injection, surgeons typically wait about 20 min for the haemostatic effect of epinephrine before incision. The flexor tendon repair will be performed as per standard practice using 4-strand core repair with epitendinous stitch as per the British Society for Surgery of the Hand (BSSH) guidelines.^27^

#### General/regional anaesthesia

Patients will have the procedure in an operating theatre under either RA or GA as per the anaesthetist’s safe standard practice. RA is preferentially chosen unless there is a contraindication. An axillary, supraclavicular or infraclavicular brachial plexus block is performed awake using ultrasound guidance and may be supplemented with ultrasound guided peripheral nerve (ulnar and/or median) blocks. Lignocaine (1% or 2%) and/or bupivacaine (0.25% or 0.5%) with or without 1:200 000 epinephrine is given for the blocks at a calculated safe dose depending on the patient’s weight. GA is administered either as inhalational anaesthesia using sevoflurane or total intravenous anaesthesia with propofol and a short-acting opiate (remifentanil or alfentanil). Analgesia is given intravenously as per the WHO analgesic ladder during a GA, with LA topical infiltration by the surgeons. The surgical procedure will be performed as per standard practice with the use of an arm tourniquet for haemostasis. The tendon repair will be performed with 4-strand core repair with epitendinous stitch as per BSSH guidelines.^27^

#### Postoperative care

All patients will have a hand therapy appointment within 3–5 days of the operation to commence early active motion and have dressings changed (as per the BSSH guidelines).^27^ All patients will have follow-up appointments with the hand therapy team for ongoing rehabilitation and dressing changes every 5–7 days until the wound heals. They will then have follow-up appointments every 5–7 days for 1 month as clinically required.

### Study outcomes

#### Primary outcome and measures

The primary outcome of the trial will be the ability to recruit patients at the selected sites (recruitment rate). Feasibility will be monitored through screening and randomisation logs with the aim to recruit 1 to 2 participants per month per site. Advisory group meetings involving patients, clinicians and methodologists will explore the following:

- Equipoise and willingness of clinicians to recruit and patients to participate.
- Withdrawals from the trial or failure to complete the follow-up visits.
- The optimal primary outcome for subsequent definitive trial on completion of the trial and presentation of the data (based on clinical and qualitative data).

The primary outcome measures are:

1. Recruitment into a trial of this nature (recruitment rate at each centre over 16 months).
2. Support for the trial from involved clinicians (assessed through qualitative interviews).
3. Equipoise among clinicians and patients (assessed through qualitative interviews).
4. Rate of withdrawal/drop-out from the study (assessed throughout the trial).

#### Secondary outcome and measures

Our secondary outcome is to determine the primary outcome and outcome measures for a subsequent definitive RCT. Several secondary outcome measures will be collected in order to help with the design of a definitive trial to determine the effectiveness and cost-effectiveness of performing flexor tendon repair under WALANT versus RA/GA. The secondary outcome measures are:

1. Difference in the proportion of good functional outcome measured using Total Active Motion (TAM) Score at 1, 3 and 6 months. The TAM Score measures the degree of movements of the finger joints using a goniometer, an instrument which measures an angle and compares it against the uninjured contralateral finger which gives percentage of normal. The results more than 75% is classified as good functional outcome.
2. Difference in grip strength and pinch strength at 1, 3 and 6 months.
3. Difference in postoperative finger oedema—measured at 1, 3 and 6 months.
4. Patient-reported outcome at 1, 3 and 6 months using the Michigan Hand Outcomes Questionnaire (MHQ) (patient-reported questionnaire on interference with daily life measured on a 5-point Likert scale involving six domains).^28^
5. Productivity impact and health-related quality of life assessed at baseline, 1, 3 and 6 months using the Work Productivity and Activity Impairment Questionnaire: Specific Health Problem and EuroQol 5 Dimensions 5 Levels instrument (EQ-5D-5L) questionnaires.^29 30^
6. Pain-assessment using Visual Analogue Score tool, which uses a 10-point Likert scale to assess pain immediately post surgery.
7. Difference in proportion of patients with tendon ruptures following repair at 1, 3 and 6 months.
8. Difference in proportion of patients with tenolysis at 6 months.
9. Difference in proportion of patients with overall complications at 6 months.
10. Adverse events.
11. Healthcare resource use.

### Participant timeline

**Figure 1:**
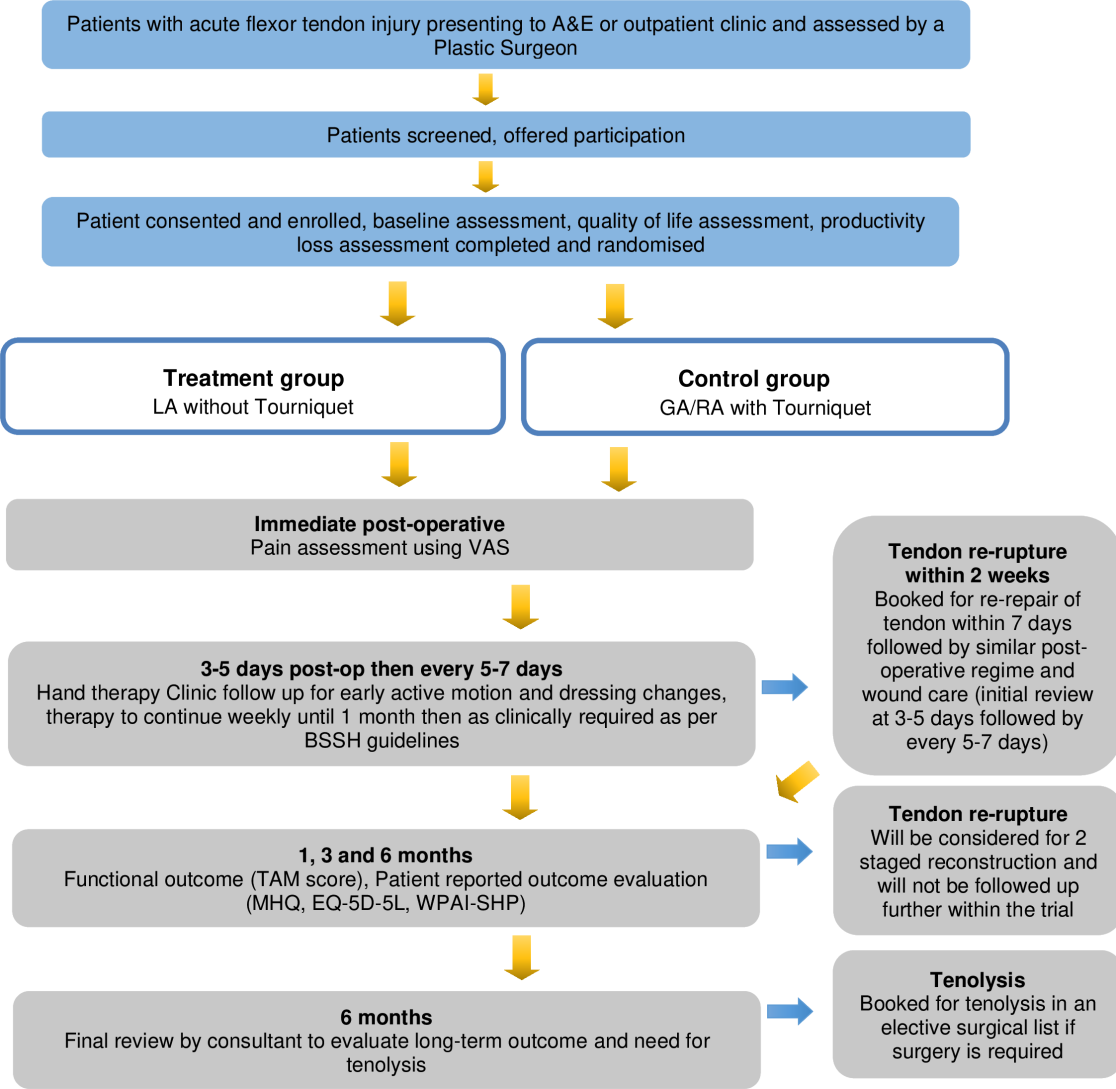
Flow chart on patient’s journey throughout the study. A&E, accident and emergency; BSSH, British Society for Surgery of the Hand; EQ-5D-5L, EuroQol 5 Dimensions 5 Levels instrument; GA, general anaesthesia; LA, local anaesthesia; MHQ, Michigan Hand Outcomes Questionnaire; RA, regional anaesthesia; TAM, Total Active Motion; VAS, Visual Analogue Score; WPAI-SHP, Work Productivity and Activity Impairment Questionnaire: Specific Health Problem.

The study was opened for recruitment in March 2023 and is anticipated to close in September 2024. Each patient will be followed up for 6 months. Figure 1 summarises patient’s journey throughout the trial.

### Sample size

A sample size of 200 eligible patients will be approached and asked to give their consent to be randomised into the trial. We anticipate that the approximate recruitment (randomisation) rate will be 30% of the approached patients. As such, 200 patients would be sufficient to be able to estimate the consent rate to within±0.07 through construction of 95% CI for the recruitment rate. Under the assumption that the recruitment rate is approximately 30%, we anticipate that up to 60 patients will be randomised (1:1 WALANT:RA/GA) to take part in the study. Our local audit informed that on an average over 100 cases of tendon repairs are performed per year by each centre of varying complexity.^31^

### Randomisation

Patients who meet the eligibility criteria will be randomised to have flexor tendon repair under GA/RA or WALANT on a 1:1 ratio. Randomisation will be performed by a study researcher using REDCap. Participants will be deemed enrolled into the study once they have been randomised to either of the study arms.

### Blinding

The surgical team, clinical staff and patient will not be blinded to the randomised intervention status. The hand therapists assessing the outcomes during follow-up visits will be blinded to the allocation.

### Data collection and management

Data will be collected at the initial assessment, intraoperatively and at follow-up assessments. Data collection is completed by trained surgeons and hand therapists using a paper ‘clinical research form’ (CRF). A local data manager at each site will then enter the data from the CRF’s onto REDCap electronic data tools database. Completeness of data will be ensured by reviewing the patient’s medical notes. A central data manager will ensure the accuracy of the data collection by preforming sample assessments at regular intervals. Any adverse events will be recorded and reported to the primary investigators and institutional ethics committee.

### Qualitative assessments

We will conduct semistructured interviews informed by a topic guide developed from the existing literature in conjunction with the trial management group, which includes patient representatives. We will explore the reasons for participation and non-participation of patients and the acceptability of the trial to clinicians and patients to assist in improvement of recruitment strategies employed for the definitive trial. The interview will be audio recorded, but the researcher will also take notes. These notes will be summarised and added to a Rapid Research, Evaluation and Appraisal Lab (RREAL) Sheet per study site so emerging findings can be shared with the trial team as the qualitative evaluation is ongoing.^32 33^

Participants will be divided into two groups according to whether they agreed (including cases of withdrawal from the trial) or did not agree to be randomised to the trial. The interviews will explore patient’s perspectives of the treatment, their understanding of the two treatments, their reasons for taking part or refusing the trial and their acceptability of randomisation between the procedures. Purposive sampling will be used to select participants according to their age, pre-existing comorbidities and recruitment centre. A total of 40 patient interviews and 15 staff interviews will be conducted across the 3 sites. This method of sampling will ensure that a wide range of experiences and perceptions are collected. Interviews will continue until data saturation is reached. We will recruit patients who refused randomisation, those who consented and those who withdrew consent. We will also recruit members of staff in charge of delivering the trial to understand trial delivery processes and identify any issues with recruitment. Recruitment to the interviews will take place alongside recruitment to the trial.

### Data storage

The data extracted for the purposes of this study will be anonymised. All handling, processing and storage of personal identifiable data and study data will be in accordance with the Data Protection Act 1998 and the NHS Code of Confidentiality.

### Statistical analysis

#### Quantitative analysis

The baseline characteristics of the two groups will be reported using means and SD or medians and IQRs for continuous variables, as appropriate, and frequency counts and percentages for categorical variables. The proportion of patients who consent to be randomised will be presented with a 95% CI. For other outcomes, we will explore the difference in proportion (for binary outcomes) and mean or median difference (for continuous outcomes) between the two groups will be presented with associated 95% CIs. No formal comparisons between the groups will be made and no hypothesis tests will be carried out. The results will inform us how sensitive the outcome measures are and, along with other information, will be used to determine the primary outcome of a subsequent large RCT. The results will also inform a sample size calculation for the primary outcome chosen.

#### Qualitative analysis

Interviews will be transcribed verbatim and coded using NVivo Computer Assisted Qualitative Data Analysis Software. The transcripts will then be analysed using framework analysis.^34^ The transcripts will be analysed by two researchers. The first researcher will fully code and analyse all transcripts. A sample of these will then be analysed by a second researcher. The main themes to include in the framework will be double-checked by the second researcher, and any disagreements will be discussed and resolved within the research team. Information gained from the qualitative process evaluation will be used to inform the development of the protocol for the definitive trial. This may mean the development of training materials for staff, changes to participant information sheets, changes to recruitment processes, etc.

#### Health economic analysis

This feasibility study will be used to plan the evaluation within the subsequent trial, which will aim to estimate the cost and the potential benefit in economic terms from the NHS perspective and personal social services. We will also explore the feasibility of collecting data to assess the costs for patients and families from a broader (societal) perspective.

Within the feasibility study of economic evaluation, we will:

1. Perform a literature search to identify economic studies and evidence available on similar interventions (eg, to capture cost-effectiveness models and data that might be needed in addition to those collected within the trial).
2. Identify, in consultation with clinicians, the main cost components of WALANT, GA and RA (equipment, materials, staff cost).
3. Identify the resource use and unit cost data for each of these components.
4. Identify the potential costs of treating side effects in both treatments.
5. Identify potential instruments to estimate patient-reported outcome and quality of life in patients in addition to MHQ and EQ-5D-5L questionnaires.
6. Identify the main costs for patients and families (eg, transport costs, caregivers’ costs, productivity losses) and best tools to collect the data.
7. Design a Markov model using data from the feasibility study to describe the patients’ pathway in the two treatment arms and to model costs and effects beyond trial, assuming a life-time horizon (the model data will be chosen from up-to-date UK sources, published literature and clinicians and patient and public representatives).

With the available data, we will perform a preliminary analysis of the cost-utility of WALANT against RA/GA for flexor tendon repair to inform the future trial.

## Discussion

The purpose of this study is to determine the feasibility of conducting an RCT that compares the use of WALANT and RA/GA as methods of anaesthesia for flexor tendon repairs. The results of this study will inform the design of a larger, more comprehensive trial. By examining factors such as patient and clinician acceptability, recruitment rates and key clinical outcome measures, the study aims to identify the most important and relevant considerations for a definitive trial. Additionally, the study will also evaluate the cost-effectiveness of WALANT as compared with the traditional standard of care in order to provide insights into the financial implications of using this method of anaesthesia.

In designing this study, we conducted a retrospective observational study, an online survey and performed a focus group meeting with experts in the field, including hand surgeons, hand therapists and patients. In our retrospective study, we examined 151 patients involving a total of 251 tendon ruptures repaired either under WALANT or Regional/GA anaesthesia.^35^ Our analysis revealed no significant differences in rates of tendon rupture, adhesions, infection or hand function between the two groups. These findings suggest that WALANT may be a safe and effective alternative to GA/RA with comparable surgical and functional outcomes. Additionally, WALANT allowed for intraoperative testing of the repair, provided patient education and had the potential to increase theatre efficiency and cost-effectiveness. However, this data is limited by the retrospective nature of the study, small sample size and potential confounders related to severity of injury, therefore the true difference between groups is not known. Meanwhile, an online survey involving 60 participants (26 hand surgeons, 7 hand therapists and 27 patients) followed by a focus group meeting involving 22 participants (8 hand surgeons, 2 hand therapists and 12 patients) were carried out to identify priorities and outcomes of a flexor tendon study. Most of our survey participants (95.3%) agreed that a trial comparing WALANT against RA/GA should be performed. Overall, 83.9% of clinicians were willing to recruit or encourage recruitment, while 83% of patients were willing to participate in such a study. The focus group participants agreed that the important outcomes that should be compared are functional outcome, patient satisfaction and the difference in complications. The patients in our focus group mentioned that they would consider the new approach only if it provided at least equivalent hand function as the current standard of care. Our survey and focus group indicated good support from clinicians and patients to participate in a trial of this nature; however, there is a need to evaluate equipoise among all parties. Our focus group also suggested that some outcomes are more important than others. Therefore, it is important to find out the acceptability of the trial to patients and clinicians, recruitment rate, primary outcome of subsequent definitive trial and the difference in the primary outcome that can be considered a meaningful difference. Therefore, a feasibility study needs to be conducted to assess the practicality of conducting a larger, more conclusive trial, as well as to determine the most important outcomes to measure. This feasibility study will also provide valuable insights for future research in hand surgery, particularly with regard to study design and outcome selection.

## Ethics and dissemination

This trial has approval from the London—City and East Research Ethics Committee (project ID: 22/PR/1197). This trial will be conducted in accordance with the Declaration of Helsinki and the recommendations of good clinical practice. The trial has been registered in ISRCTN registry (ISRCTN identifier: 15052559).

The results of the study will be reported and disseminated in peer-reviewed scientific journals, conference presentations and website/online publications, as well as in internal reports. Reporting will be based on Consolidated Standards of Reporting Trials guideline for reporting randomised trials. All publications will be forwarded to participants.

### Trial status

This trial has begun recruitment in March 2023.

## Supplementary Material

Reviewer comments

Author's manuscript
