# Supplementary material for: WAFER trial: a study protocol for a feasibility randomised controlled trial comparing wide-awake local anaesthesia no tourniquet (WALANT) to general and regional anaesthesia with tourniquet for flexor tendon repair
Source: BMJ Open. 2023 Aug 28;13(8):e075440. doi: 10.1136/bmjopen-2023-075440 (PMC10462963; doi:10.1136/bmjopen-2023-075440)
Supplement: Author's manuscript [file bmjopen-2023-075440.draft_revisions.pdf]

BMJ Open is committed to open peer review. As part of this commitment we make the peer review history of every article we publish publicly available.

When an article is published we post the peer reviewers' comments and the authors' responses online. We also post the versions of the paper that were used during peer review. These are the versions that the peer review comments apply to.

The versions of the paper that follow are the versions that were submitted during the peer review process. They are not the versions of record or the final published versions. They should not be cited or distributed as the published version of this manuscript.

BMJ Open is an open access journal and the full, final, typeset and author-corrected version of record of the manuscript is available on our site with no access controls, subscription charges or pay-per-view fees (http://bmjopen.bmj.com).

If you have any questions on BMJ Open's open peer review process please email info.bmjopen@bmj.com

BMJ Open

WAFER Trial: A study protocol for a feasibility randomised controlled trial comparing Wide-awake Local Anaesthesia No Tourniquet (WALANT) to General and Regional Anaesthesia for Flexor Tendon Repair

Journal:	*BMJ Open*
Manuscript ID	bmjopen-2023-075440
Article Type:	Protocol
Date Submitted by the Author:	08-May-2023

Complete List of Authors:	Kanapathy, Muholan; University College London, Division of Surgery and Interventional Science ; Royal Free NHS Foundation Trust Hospital, Department of Plastic and Reconstructive Surgery<br>Faderani, Ryan; Royal Free NHS Foundation Trust Hospital, Department of Plastic and Reconstructive Surgery; University College London<br>Bray, Juliette; Royal Free NHS Foundation Trust Hospital, Department of Plastic and Reconstructive Surgery<br>Dehbi, Hakim-Moulay; University College London Institute of Clinical Trials and Methodology<br>Panca, Monica; University College London, Comprehensive Clinical Trials Unit<br>Vindrola-Padros, Cecilia; University College London, Department of Targeted Intervention<br>Prasad, Anjana; Royal Free NHS Foundation Trust Hospital, Department of Anaesthesia<br>Burr, Nicola; Royal Free NHS Foundation Trust Hospital, Department of Plastic and Reconstructive Surgery<br>Williams, Norman; University College London, UCL Division of Surgery & Interventional Science<br>Bhat, Waseem; Leeds Teaching Hospitals NHS Trust, Department of Plastic and Reconstructive Surgery<br>Wong, Jason ; The University of Manchester Faculty of Biology Medicine and Health, Blond McIndoe Laboratories, Division of Cell Matrix Biology and Regenerative Medicine<br>Mosahebi, Afshin; University College London Division of Surgery and Interventional Science; Royal Free NHS Foundation Trust Hospital, Department of Plastic and Reconstructive Surgery<br>Nikkhah, Dariush; Royal Free NHS Foundation Trust Hospital, Department of Plastic and Reconstructive Surgery; University College London

Keywords:	ANAESTHETICS, Anaesthesia in orthopaedics < ANAESTHETICS, Hand & wrist < ORTHOPAEDIC & TRAUMA SURGERY, PLASTIC & RECONSTRUCTIVE SURGERY, Plastic & reconstructive surgery < SURGERY

SCHOLARONE™
Manuscripts

Title Page

Title:

WAFER Trial: A study protocol for a feasibility randomised controlled trial comparing Wide-awake Local Anaesthesia No Tourniquet (WALANT) to General and Regional Anaesthesia for Flexor Tendon Repair

For peer review only

Authors:

Mr Muholan Kanapathy MD MRCS MSc PhD [1, 2]

Mr Ryan Faderani MBBS BSc MRCS FHEA [1, 3]

Miss Juliette Bray BSc (Hons) [1]

Dr Hakim-Moulay Dehbi PhD [4]

Miss Monica Panca MSc [4]

Prof Cecilia Vindrola-Padros BA MA PhD [5]

Dr Anjana Prasad BMedSci (Hons) BMBS FRCA [6]

Miss Nicola Burr Grad Dip Phys MSc [2]

Prof Norman Williams BSc PhD CSci MICR FIBMS [2]

Mr Waseem Bhat MD MA MSc FRCS, Dipl Hand Surg [7]

Mr Jason Wong MBChB FHEA PhD FRCS (plast) [8,9]

Prof Afshin Mosahebi MBBS MBA PhD FRCS (plast) [1,2] *

Mr Dariush Nikkhah BM MSc FRCS (plast) [1,2,3] *

* Co-senior Authors

Affiliations:

[1]Department of Plastic and Reconstructive Surgery, Royal Free NHS Foundation Trust Hospital, London, United Kingdom

[2] Division of Surgery & Interventional Science, University College London, United Kingdom

[3] Faculty of Medical sciences, University College London, United Kingdom

[4] Comprehensive Clinical Trials Unit, University College London, United Kingdom

[5] Department of Targeted Intervention, University College London, United Kingdom

[6] Department of Anaesthesia, Royal Free NHS Foundation Trust Hospital, London, United Kingdom

[7] Department of Plastic and Reconstructive Surgery, Leeds Teaching Hospitals NHS trust, Leeds, United Kingdom

[8] Department of Plastic and Reconstructive Surgery, Manchester University NHS Foundation Trust, Manchester, United Kingdom

[9] Blond McIndoe Laboratories, Division of Cell Matrix Biology and Regenerative Medicine, Manchester Academic Health Science Centre, School of Biological Sciences, Faculty of Biology, Medicine and Health, The University of Manchester, Manchester, United Kingdom

Corresponding author:

Mr Ryan Faderani

Department of Plastic and Reconstructive Surgery, Royal Free NHS Foundation Trust Hospital, London, United Kingdom

Email: Ryan.faderani.13@ucl.ac.uk

Protocol Version: 1.2 8/2/2023

Key words:

Tendon, Flexor tendon, Hand Surgery, Anaesthesia, WALANT

Word Count: 3871

For peer review only

Abstract

Introduction:

Flexor tendons are traditionally repaired under either general anaesthesia(GA) or regional anaesthesia(RA), allowing for the use of an arm tourniquet to minimise blood loss and establish a bloodless surgical field. However, the use of tourniquets exposes the patient to certain risks, including skin, muscle, and nerve injuries. A recent advancement in anaesthesia delivery involves the use of a wide-awake approach where no sedation nor tourniquets are used (WALANT). WALANT utilises local anaesthetic with adrenaline to provide pain relief and vasoconstriction, reducing operative bleeding. Several studies revealed potential benefits for WALANT compared to GA or RA. However, there remains a paucity of high-quality evidence to support the use of WALANT. As a result of this uncertainty, the clinical practice varies considerably. We aim to evaluate the feasibility of WALANT as an alternative to GA and RA in patients undergoing surgical repair of flexor tendon injuries. This involves addressing factors such as clinician and patient support for a trial, clinical equipoise, trial recruitment and dropout, and the most relevant outcomes measures for a future definitive trial.

Methods and analysis:

WAFER is a multi-centre, single-blinded, parallel group, randomised controlled trial (RCT) to assess the feasibility of WALANT versus RA and GA. The target population is patients with acute traumatic flexor tendon injuries, across three major hand surgery units in England involving a total of 60 participants. Outcome assessors will be blinded. The primary outcome will be the ability to recruit patients into the trial, whilst secondary outcomes include difference in functional outcome, patient-reported outcome measures, health-related quality of life, cost-effectiveness, and complication rates.

Ethics and dissemination:

Ethical approval was obtained from the London – City and East Research Ethics Committee (22/PR/1197). Findings will be disseminated through peer-reviewed publication, conferences, patient information websites and social media networks.

For peer review only

Trial registration:

ISRCTN identifier:15052559

Strengths and limitations of this study

- This feasibility study will use mixed methods to assess the feasibility of conducting a randomised controlled trial exploring the effectiveness and cost-effectiveness of performing flexor tendon repair under local anaesthesia compared to general or regional anaesthesia.
- This study has the potential to identify barriers in conducting randomised controlled trials in the context of hand trauma.
- This study will also provide valuable insights for future research in hand surgery, particularly in regard to study design and outcome selection.
- One limitation of this study is its feasibility nature, which means that the sample size is not sufficient to detect differences between groups with a high degree of confidence. However, the data obtained from this study will be highly valuable in designing a subsequent definitive trial.

For peer review only

Introduction

Hand injuries account for up to 20% of all presentations to emergency departments, equating to 4.6 million patients annually in the United Kingdom alone, and resulting in an average of 8 weeks of lost workdays (1, 2). In patients with hand injuries, tendon lacerations are common and are reported in 54.8% of patients with a shallow laceration and 92.5% with deeper injury (3). Patients with complete tendon laceration will require surgical repair, which is typically performed in the operating room, using an arm tourniquet to minimise blood loss and provide a clear surgical field (4). The use of a tourniquet is associated with significant discomfort and is not well tolerated by patients without general anaesthesia (GA) or regional anaesthesia (RA) (5).

A recent advancement in the method of anaesthesia delivery in hand surgery involves the introduction of Wide-awake Local Anaesthesia No Tourniquet (WALANT) (6,7,8). WALANT involves administering local anaesthesia (LA) with adrenaline for anaesthetic and vasoconstriction effect respectively (8). The vasoconstrictive effect of adrenaline achieves reduced bleeding at the site of surgery, hence acting as a chemical tourniquet and eliminating the need for a mechanical arm tourniquet. Removing the need for tourniquet and therefore GA/RA has several potential advantages for patients. Firstly, patients can make immediate post-operative recovery, ultimately shortening the patient stay in hospital. GA is associated with side effects such as nausea and vomiting along with serious cardiac and respiratory risks especially amongst patients undergoing emergency surgery, elderly, smokers, and overweight patients (9). RA avoids these risks but exposes the patient to the risk of permanent or temporary nerve damage (1 in 10) and failure of providing adequate pain relief resulting in conversion to GA (3 in 100) (10, 11). Tourniquet use has also been linked to increased post-operative pain and side effects such as muscle, nerve and skin injury, and post-tourniquet thrombosis (5, 12).

While WALANT requires multiple injections that may cause temporary discomfort, it avoids many of the significant issues associated with GA and RA. Furthermore, patients who undergo WALANT have been reported to have a decreased need for post-operative pain relief (13). Additionally, WALANT allows the surgeon to intraoperatively evaluate the tendon repair while the patient actively moves their fingers, allowing adjustments to be made before skin closure.

This has been shown to improve surgical outcomes and reduce the need for revision surgery (14, 15). Patients can also receive education during the procedure to aid their rehabilitation (7). There are also potential economic and environmental benefits to using WALANT, as it does not require an anaesthetist, requires fewer clinical staff (anaesthetist, anaesthetic assistant, recovery nurse), and produces fewer anaesthetic waste gases (16, 17).

Rationale

In recent years, WALANT has gained significant popularity as an alternative to GA or RA with tourniquet for flexor tendon repairs (18). While studies have suggested that it may have similar safety and potential benefits in comparison to GA or RA with a tourniquet, it should be noted that these studies are either retrospective in nature or lack sufficient statistical power to make definitive conclusions (19 – 22). Furthermore, it is important to determine the outcomes that are important to be measured, evaluate equipoise amongst clinician to recruit to a study comparing WALANT against GA/RA, and acceptability of the trial to patients and clinician before a definitive trial can be carried out. In addition, because of the uncertainty about the effectiveness and cost-effectiveness of WALANT compared to standard of care and the variation is clinical practice, a definitive RCT on the effectiveness and cost-effectiveness of WALANT is necessary. A feasibility study is hence necessary to help with the design of the definitive RCT. The WAFER trial therefore aims to evaluate the feasibility of WALANT as an alternative to GA or RA in patients undergoing surgical repair of traumatic laceration of flexor tendons of the hand.

For peer review only

Methods and analysis

Trial design

The WAFER trial is a multicentre, assessor-blinded, feasibility RCT with 2 parallel groups (WALANT vs. GA/RA) conducted in 3 sites within the UK. Eligible patients will be randomised to WALANT or GA/RA for flexor tendon repair using a computerised randomisation method. This protocol is reported in accordance with SPIRIT 2013 guidelines (23).

Patient and public involvement

Patient representatives who have had flexor tendon injury were involved in the design of this trial by providing feedback via online surveys and focus group meetings. A total of 27 patients were involved in the online survey and another 12 patients were involved in focus group meetings to identify priorities and outcomes of a flexor tendon study. Two patient representatives have been involved in designing and revising the trial to ensure that patient interests are central to the study design and that the trial is written clearly avoiding excessive or unnecessary medical jargon.

Study setting

Participants will be recruited at the Royal Free Hospital, Manchester University NHS Foundation Trust, and Leeds Teaching Hospital NHS Trust. This trial is open to recruiting additional sites through NIHR Clinical Research Network (CRN).

Eligibility criteria

Patients presenting to the Accident and Emergency (A&E) unit or outpatient clinics with acute traumatic flexor tendon ruptures will be reviewed by a hand surgeon and assessed. Normal clinical practice is that all patients will be assessed clinically and if the flexor tendon is lacerated, it will be scheduled for repair under GA/RA or WALANT in the operating theatre. Upon scheduling the patient for surgery, patients will be flagged to the research team. At this time, patients will be screened and where appropriate be offered a patient information sheet for inclusion into the trial. This process will include explanation of the aims, methods of

For peer review only - http://bmjopen.bmj.com/site/about/guidelines.xhtml

anaesthesia and surgery, anticipated benefits, and potential hazards of the study. Patients will be given sufficient time (24 hours or more if needed) to consider whether they wish to participate. Once the patients are ready for surgery, following review by the study team, patients will be assessed clinically to confirm 'inclusion and exclusion criteria' are met and informed consent form will be completed, following which patients will be randomised to either of the treatment arm and enrolled into the study. Patient related data will be recorded in a Patient Assessment Form.

Inclusion criteria

1. Adults ≥ 18 years old.
2. Clinical diagnosis of acute flexor tendon injury within 2 weeks of injury (≤2 weeks from injury to date of surgery).
3. Complete laceration (100% transection) of Flexor digitorum profundus, and/or Flexor digitorum superficialis, and/or Flexor pollicis longus of the hand.
4. Single digit or two digits injury involving flexor zone 1 to 3.
5. Patient understands and is willing to participate and can comply with the follow-up regime.

Exclusion criteria

1. Tendon not amendable to primary repair (gross wound contamination, segmental tendon loss, associated fractures, or mangled hand injuries).
2. Secondary tendon repair or reconstruction.
3. History of allergy to LA.
4. Refusal to have LA or deemed non-cooperative to be performed without sedation.
5. Pre-existing deformity of finger or hand.
6. High risk for GA or not fit for surgery (ASA ≥ 4).

Interventions

WALANT

For peer review only

Patients will have the procedure in an operating theatre under WALANT without an arm tourniquet. WALANT uses a mixture of local anaesthesia with adrenaline to provide anaesthesia and haemostasis. A mixture of 1% xylocaine with adrenaline 1:200,000 will be injected into the digit or hand requiring surgery. This mixture is routinely used in clinical practice (24, 25). The mixture is used in a "field block" technique where 2ml is injected in the subcutaneous fat in the middle of the base of the proximal and middle phalanges between both digital nerves, and 1 ml is injected in the distal phalanx. In addition, 10- 15ml local anaesthesia will be injected in the palm where dissection will be performed (26).

Following injection, surgeons typically wait about 20 minutes for the haemostatic effect of adrenaline before incision. The flexor tendon repair will be performed as per standard practice using 4-strand core repair with epitendinous stitch as per the British Society for Surgery of the Hand (BSSH) guidelines (27).

General / Regional anaesthesia

Patients will have the procedure in an operating theatre under either RA or GA as per the anaesthetist's safe standard practice. RA is preferentially chosen unless there is a contra-indication. An axillary, supraclavicular or infraclavicular brachial plexus block is performed awake using ultrasound guidance and may be supplemented with ultrasound guided peripheral nerve (ulnar and/or median) blocks. Lignocaine (1% or 2%) and/or Bupivacaine (0.25% or 0.5%) with or without 1:200,000 adrenaline is given for the blocks at a calculated safe dose depending on the patient's weight. GA is administered either as inhalational anaesthesia using sevoflurane or total intravenous anaesthesia with propofol and a short-acting opiate (remifentanil or alfentanil). Analgesia is given intravenously as per the World Health Organisation analgesic ladder during a GA, with LA topical infiltration by the surgeons. The surgical procedure will be performed as per standard practice with the use of an arm tourniquet for haemostasis. The tendon repair will be performed with 4-strand core repair with epitendinous stitch as per BSSH guidelines (27).

Post-operative care

All patients will have a hand therapy appointment within 3-5 days of the operation to commence early active motion and have dressings changed (as per the British Society for

For peer review only

Surgery of the Hand guidelines) (27). All patients will have follow-up appointments with the hand therapy team for ongoing rehabilitation and dressing changes every 5-7 days until the wound heals. They will then have follow-up appointments every 5-7 days for 1 month as clinically required.

Study outcomes:

Primary outcome & measures

The primary outcome of the trial will be the ability to recruit patients at the selected sites (recruitment rate). Feasibility will be monitored through screening and randomisation logs with the aim to recruit 1 to 2 participants per month per site. Advisory group meetings involving patients, clinicians and methodologist will explore the following:

- equipoise and willingness of clinicians to recruit and patients to participate.
- withdrawals from the trial or failure to complete the follow-up visits.
- the optimal primary outcome for subsequent definitive trial on completion of the trial and presentation of the data (based on clinical and qualitative data).

The primary outcome measures are:

1. Recruitment into a trial of this nature (recruitment rate at each centre over 16 months)
2. Support for the trial from involved clinicians (Assessed through qualitative interviews)
3. Equipoise amongst clinicians and patients (Assessed through qualitative interviews)
4. Rate of withdrawal/drop-out from the study (Assessed throughout the trial)

Secondary outcome & measures

Our secondary outcome is to determine the primary outcome and outcome measures for a subsequent definitive randomised controlled trial. Several secondary outcome measures will be collected in order to help with the design of a definitive trial to determine the effectiveness and cost-effectiveness of performing flexor tendon repair under WALANT vs RA/GA. The secondary outcome measures are:

For peer review only

1. Difference in the proportion of good functional outcome measured using Total Active Motion (TAM) score at 1, 3 and 6 months. The TAM score measures the degree of movements of the finger joints using a goniometer, an instrument which measures an angle, and compares it against the uninjured contralateral finger which gives percentage of normal. Results more than 75% is classified as good functional outcome.
2. Difference in grip strength and pinch strength at 1, 3, and 6 months.
3. Difference in post-operative finger oedema – measured at 1, 3, and 6 months.
4. Patient reported outcome at 1, 3 and 6 months using the Michigan Hand Outcomes Questionnaire (MHQ) (patient-reported questionnaire on interference with daily life measured on a 5-point Likert scale involving 6 domains). (28)
5. Productivity impact and health-related quality of life-assessed at baseline, 1, 3 and 6 months using the Work Productivity and Activity Impairment Questionnaire: Specific Health Problem (WPAI-SHP) and EQ-5D-5L (evaluates 5 main health domains in 5 levels of severity) questionnaires. (29, 30)
6. Pain-assessment using Visual analogue score (VAS) tool, which uses a 10-point Likert scale to assess pain immediately post-surgery.
7. Difference in proportion of patients with tendon ruptures following repair at 1, 3 and 6 months.
8. Difference in proportion of patients with tenolysis at 6 months.
9. Difference in proportion of patients with overall complications at 6 months.
10. Adverse events.
11. Healthcare resource use.

Participant timeline

The study was opened for recruitment in March 2023 and is anticipated to close in September 2024. Each patient will be followed up for 6 months. Figure 1 summarises patient's journey throughout the trial.

For peer review only

Sample size

A sample size of 200 eligible patients will be approached and asked to give their consent to be randomised into the trial. We anticipate that the approximate recruitment (randomisation) rate will be 30% of the approached patients. As such, 200 patients would be sufficient to be able to estimate the consent rate to within ± 0.07 through construction of 95% confidence interval for the recruitment rate. Under the assumption that the recruitment rate is approximately 30%, we anticipate that up to 60 patients will be randomised (1:1 WALANT:RA/GA) to take part in the study. Our local audit informed that on average over 100 cases of tendon repairs are performed per year by each centre of varying complexity (31).

Randomisation

Patients who meet the eligibility criteria will be randomised to have flexor tendon repair under GA/RA or WALANT on a 1:1 ratio. Randomisation will be performed by a study researcher using REDCap. Participants will be deemed enrolled into the study once they have been randomised to either of the study arms.

Blinding

The surgical team, clinical staff, and patient will not be blinded to the randomised intervention status. The hand therapists assessing the outcomes during follow-up visits will be blinded to the allocation.

Data collection and management

Data will be collected at the initial assessment, intra-operatively and at follow-up assessments. Data collection is completed by trained surgeons and hand therapists using paper 'clinical research form '(CRF). A local data manager at each site will then enter the data from the CRF's onto REDCap electronic data tools database. Completeness of data will be ensured by reviewing the patient's medical notes. A central data manager will ensure the accuracy of the data collection by preforming sample assessments at regular intervals. Any adverse events will be recorded and reported to the primary investigators and institutional ethics committee.

For peer review only

Qualitative assessments

We will conduct semi-structured interviews informed by a topic guide developed from the existing literature in conjunction with the trial management group, which includes patient representatives. We will explore the reasons for participation and non-participation of patients and the acceptability of the trial to clinicians and patients to assist in improvement of recruitment strategies employed for the definitive trial. The interview will be audio recorded, but the researcher will also take notes. These notes will be summarised and added to a RREAL Sheet per study site so emerging findings can be shared with the trial team as the qualitative evaluation is ongoing (32,33).

Participants will be divided into two groups according to whether they agreed (including cases of withdrawal from the trial) or did not agree to be randomised to the trial. The interviews will explore patient's perspectives of the treatment, their understanding of the two treatments, their reasons for taking part or refusing the trial, and their acceptability of randomisation between the procedures. Purposive sampling will be used to select participants according to their age, pre-existing comorbidities, and recruitment centre. A total of 40 patient interviews and 15 staff interviews will be conducted across the 3 sites. This method of sampling will ensure that a wide range of experiences and perceptions are collected. Interviews will continue until data saturation is reached. We will recruit patients who refused randomisation, those who consented, and those who withdrew consent. We will also recruit members of staff in charge of delivering the trial to understand trial delivery processes and identify any issues with recruitment. Recruitment to the interviews will take place alongside recruitment to the trial.

Data storage

The data extracted for the purposes of this study will be anonymised. All handling, processing, and storage of personal identifiable data and study data will be in accordance with the Data Protection Act 1998 and the NHS Code of Confidentiality.

For peer review only

Statistical analysis

Quantitative analysis:

The baseline characteristics of the two groups will be reported using means and standard deviations or medians and inter-quartile ranges for continuous variables, as appropriate, and frequency counts and percentages for categorical variables. The proportion of patients who consent to be randomised will be presented with a 95% confidence interval. For other outcomes, we will explore the difference in proportion (for binary outcomes) and mean or median difference (for continuous outcomes) between the two groups will be presented with associated 95% confidence intervals. No formal comparisons between the groups will be made and no hypothesis tests will be carried out. The results will inform us how sensitive the outcome measures are and, along with other information, will be used to determine the primary outcome of a subsequent large RCT. The results will also inform a sample size calculation for the primary outcome chosen.

Qualitative analysis:

Interviews will be transcribed verbatim and coded using NVivo Computer Assisted Qualitative Data Analysis Software. The transcripts will then be analysed using framework analysis (34). The transcripts will be analysed by two researchers. The first researcher will fully code and analyse all transcripts. A sample of these will then be analysed by a second researcher. The main themes to include in the framework will be double-checked by the second researcher, and any disagreements will be discussed and resolved within the research team. Information gained from the qualitative process evaluation will be used to inform the development of the protocol for the definitive trial. This may mean the development of training materials for staff, changes to participant information sheets, changes to recruitment processes, etc.

Health economic analysis:

This feasibility study will be used to plan the evaluation within the subsequent trial, which will aim to estimate the cost and the potential benefit in economic terms from the NHS perspective and personal social services. We will also explore the feasibility of collecting data to assess the costs for patients and families from a broader (societal) perspective.

Within the feasibility study of economic evaluation, we will:

For peer review only

1. Perform a literature search to identify economic studies and evidence available on similar interventions (e.g. to capture cost-effectiveness models and data that might be needed in addition to those collected within the trial).
2. Identify, in consultation with clinicians, the main cost components of WALANT, GA and RA (equipment, materials, staff cost).
3. Identify the resource use and unit cost data for each of these components.
4. Identify the potential costs of treating side effects in both treatments.
5. Identify potential instruments to estimate patient reported outcome and quality of life in patients in addition to MHQ and EQ-5D-5L questionnaires.
6. Identify the main costs for patients and families (e.g. transport costs, care givers ’ costs, productivity losses) and best tools to collect the data.
7. Design a Markov model using data from the feasibility study to describe the patients pathway in the two treatment arms and to model costs and effects beyond-trial, assuming a life-time horizon (the model data will be chosen from up-to-date UK sources, published literature and clinicians and patient and public representatives

With the available data we will perform a preliminary analysis of the cost-utility of WALANT against RA/GA for flexor tendon repair to inform the future trial.

Discussion

The purpose of this study is to determine the feasibility of conducting a randomised controlled trial that compares the use of WALANT and RA/GA as methods of anaesthesia for flexor tendon repairs. The results of this study will inform the design of a larger, more comprehensive trial. By examining factors such as patient and clinician acceptability, recruitment rates, and key clinical outcome measures, the study aims to identify the most important and relevant considerations for a definitive trial. Additionally, the study will also evaluate the cost-effectiveness of WALANT as compared to the traditional standard of care in order to provide insights into the financial implications of using this method of anaesthesia.

For peer review only

In designing this study, we conducted a retrospective observational study, an online survey and performed a focus group meeting with experts in the field, including hand surgeons, hand therapists, and patients. In our retrospective study, we examined 151 patients involving a total of 251 tendon ruptures repaired either under WALANT or Regional/GA anesthesia (35). Our analysis revealed no significant differences in rates of tendon rupture, adhesions, infection, or hand function between the two groups. These findings suggest that WALANT may be a safe and effective alternative to GA/RA with comparable surgical and functional outcomes. Additionally, WALANT allowed for intraoperative testing of the repair, provided patient education, and had the potential to increase theatre efficiency and cost-effectiveness. However, this data is limited by the retrospective nature of the study, small sample size and potential confounders related to severity of injury, therefore the true difference between groups is not known. Meanwhile, an online survey involving 60 participants (26 hand surgeons, 7 hand therapists and 27 patients) followed by a focus group meeting involving 22 participants (8 hand surgeons, 2 hand therapist and 12 patients) were carried out to identify priorities and outcomes of a flexor tendon study. Most of our survey participants (95.3%) agreed that a trial comparing WALANT against RA/GA should be performed. 83.9% of clinicians were willing to recruit or encourage recruitment, while 83% of patients were willing to participate in such a study. The focus group participants agreed that the important outcomes that should be compared are functional outcome, patient satisfaction, and the difference in complications. The patients in our focus group mentioned that they would consider the new approach only if it provided at least equivalent hand function as the current standard of care. Our survey and focus group indicated good support from clinicians and patients to participate in a trial of this nature, however there is a need to evaluate equipoise amongst all parties. Our focus group also suggested that some outcomes are more important than others. Therefore, it is important to find out the acceptability of the trial to patients and clinicians, recruitment rate, primary outcome of subsequent definitive trial, and the difference in the primary outcome that can be considered a meaningful difference. Therefore, a feasibility study needs to be conducted to assess the practicality of conducting a larger, more conclusive trial, as well as to determine the most important outcomes to measure. This feasibility study will also provide valuable insights for future research in hand surgery, particularly with regards to study design and outcome selection.

For peer review only

Ethics and dissemination

This trial has approval from the London – City and East Research Ethics Committee (project ID: 22/PR/1197). This trial will be conducted in accordance with the Declaration of Helsinki and the recommendations of Good Clinical Practice. The trial has been registered in ISRCTN registry (ISRCTN identifier:15052559).

The results of the study will be reported and disseminated in peer-reviewed scientific journals, conference presentations, and website/online publications, as well as in internal reports. Reporting will be based on CONSORT guideline for reporting randomised trials. All publications will be forwarded to participants.

Trial status

This trial has begun recruitment in March 2023.

For peer review only

References

1. Wong JY. Time off work in hand injury patients. The Journal of hand surgery. 2008;33(5):718-25.

2. BSSH. Hand Surgery in the UK. Report of a Working Party. 2017. Available at: https://www.bssh.ac.uk/professionals/hand_surgery_in_the_uk.aspx. Accessed on: 25/10/2021.

3. Tuncali D, Yavuz N, Terzioglu A, Aslan G. The rate of upper-extremity deep-structure injuries through small penetrating lacerations. Annals of plastic surgery. 2005;55(2):146-8.

4. Gibson PD, Sobol GL, Ahmed IH. Zone II Flexor Tendon Repairs in the United States: Trends in Current Management. The Journal of hand surgery. 2017;42(2):e99-e108.

5. Noordin S, McEwen JA, Kragh JF, Jr., Eisen A, Masri BA. Surgical tourniquets in orthopaedics. The Journal of bone and joint surgery American volume. 2009;91(12):2958-67.

6. Nikkhah D, Ruston J, Pearl R, Blair J. 'WALANT carpal tunnel release: Technical considerations and pain outcomes'. Journal of plastic, reconstructive & aesthetic surgery : JPRAS. 2019;72(9):1576-606.

7. Lalonde D, Bell M, Benoit P, Sparkes G, Denkler K, Chang P. A multicenter prospective study of 3,110 consecutive cases of elective epinephrine use in the fingers and hand: the Dalhousie Project clinical phase. The Journal of hand surgery. 2005;30(5):1061-7.

8. Lalonde DH, Wong A. Dosage of local anesthesia in wide awake hand surgery. The Journal of hand surgery. 2013;38(10):2025-8.

9. National Health Service. Anaesthesia. Available at: https://www.nhs.uk/conditions/anaesthesia/. Accessed on 25/10/2021. NHS Evidence, 2018

10. Jeng CL, Torrillo TM, Rosenblatt MA. Complications of peripheral nerve blocks. British journal of anaesthesia. 2010;105 Suppl 1:i97-107.

11. Teunkens A, Vermeulen K, Belmans A, Degreef I, Van de Velde M, Rex S. Patient satisfaction with intravenous regional anaesthesia or an axillary block for minor ambulatory hand surgery: A randomised controlled study. European journal of anaesthesiology. 2020;37(10):847-56.

12. Gunasagaran J, Sean ES, Shivdas S, Amir S, Ahmad TS. Perceived comfort during minor hand surgeries with wide awake local anaesthesia no tourniquet (WALANT) versus local

For peer review only

anaesthesia (LA)/tourniquet. Journal of orthopaedic surgery (Hong Kong). 2017;25(3):2309499017739499.

13. Lalchandani GR, Halvorson RT, Rahgozar P, Immerman I. Wide-Awake Local Anesthesia for Minor Hand Surgery Associated With Lower Opioid Prescriptions, Morbidity, and Costs: A Nationwide Database Study. Journal of Hand Surgery Global Online. 2020;2(1):7-12.

14. Higgins A, Lalonde DH, Bell M, McKee D, Lalonde JF. Avoiding flexor tendon repair rupture with intraoperative total active movement examination. Plastic and reconstructive surgery. 2010;126(3):941-5

15. Kadhum, M., Georgiou, A., Kanapathy, M., Reissis, D., Akhavani, M., Burr, N., & Nikkhah, D. (2022). Operative outcomes for wide awake local anesthesia versus regional and general anesthesia for flexor tendon repair. Hand surgery & rehabilitation, 41(1), 125–130. https://doi.org/10.1016/j.hansur.2021.10.312

16. Yen CY, Ma CH, Wu CH, Yang SC, Jou IM, Tu YK. A cost and efficacy analysis of performing arthroscopic excision of wrist ganglions under wide-awake anaesthesia versus general anaesthesia. BMC musculoskeletal disorders. 2020;21(1):459.

17. Chatterjee A, McCarthy JE, Montagne SA, Leong K, Kerrigan CL. A cost, profit, and efficiency analysis of performing carpal tunnel surgery in the operating room versus the clinic setting in the United States. Annals of plastic surgery. 2011;66(3):245-8.

18. Søreide K, Hallet J, Matthews JB, Schnitzbauer AA, Line PD, Lai PBS, et al. Immediate and long-term impact of the COVID-19 pandemic on delivery of surgical services. The British journal of surgery. 2020

19. Reissis D, Georgiou A, Nikkhah D, O'Toole G. U.K. Response to the COVID-19 Pandemic: Managing Plastic Surgery Patients Safely. Plastic and reconstructive surgery. 2020;146(2):250e-1e.

20. Hobday D, Welman T, O'Neill N, Pahal GS. A protocol for wide awake local anaesthetic no tourniquet (WALANT) hand surgery in the context of the coronavirus disease 2019 (COVID-19) pandemic. The surgeon : journal of the Royal Colleges of Surgeons of Edinburgh and Ireland. 2020.

21. Nolan GS, Kiely AL, Madura T, Karantana A. Wide-awake local anaesthesia no tourniquet (WALANT) vs regional or general anaesthesia for flexor tendon repair in adults: protocol for a systematic review and meta-analysis. Systematic reviews. 2020;9(1):264.

For peer review only

22. Choukairi F, Ibrahim I, R NAM, Reid AJ, Winterton RI, Bedford JD, et al. Development of the Manchester wide-awake hand trauma service in 2020: the patient experience. The Journal of hand surgery, European volume. 2021;46(5):569-73.
23. Chan A-W, Tetzlaff JM, Altman DG, Dickersin K, Moher D. SPIRIT 2013: new guidance for content of clinical trial protocols. Lancet. 2013;381(9861):91–2.
24. Grandizio LC, Graham J, Klena JC. Current Trends in WALANT Surgery: A Survey of American Society for Surgery of the Hand Members. Journal of Hand Surgery Global Online. 2020;2(4):186-90.
25. Zargaran D, Zargaran A, Nikkhah D, Mosahebi A. WALANT Protocol: Stop before you block. Journal of plastic, reconstructive & aesthetic surgery : JPRAS. 2021;74(10):2776-820. (5)
26. Lalonde, D. H., & Martin, A. L. (2013). Wide-awake flexor tendon repair and early tendon mobilization in zones 1 and 2. Hand clinics, 29(2), 207–213. https://doi.org/10.1016/j.hcl.2013.02.009
27. The British Society for Surgery of the Hand, Lacerations with flexor tendon involvement guildelines, BSSH Standards of Care in Hand Trauma, https://www.bssh.ac.uk/bssh_standards_of_care_in_hand_trauma_.aspx 2020
28. Chung KC, Pillsbury MS, Walters MR, Hayward RA. Reliability and validity testing of the Michigan Hand Outcomes Questionnaire. J Hand Surg Am. 1998;23(4):575-87.
29. Reilly MC, Zbrozek AS, Dukes E: The validity and reproducibility of a work productivity and activity impairment measure. PharmacoEconomics 1993; 4(5):353-365.
30. Herdman M, Gudex C, Lloyd A, et al. Development and preliminary testing of the new five-level version of EQ-5D (EQ-5D-5L). Qual Life Res. 2011;20(10):1727-1736. doi:10.1007/s11136-011-9903-x
31. Faderani, R., Kanapathy, M., & Mosahebi, A. (2021). Hand injury: investigating the accuracy of referrals to a specialist trauma centre. Emergency nurse : the journal of the RCN Accident and Emergency Nursing Association, 29(3), 29–33. https://doi.org/10.7748/en.2021.e2065
32. Vindrola-Padros, C., Chisnall, G., Polanco, N., & San Juan, N. V. (2022). Iterative cycles in qualitative research: introducing the RREAL sheet as an innovative process. https://papers.ssrn.com/sol3/papers.cfm?abstract_id=4162797

For peer review only

33. Vindrola-Padros, C., Chisnall, G., Cooper, S., Dowrick, A., Djellouli, N., Symmons, S. M., ... & Johnson, G. A. (2020). Carrying out rapid qualitative research during a pandemic: emerging lessons from COVID-19. Qualitative health research, 30(14), 2192-2204.

34. Srivastava, A., & Thomson, S. B. (2009). Framework analysis: a qualitative methodology for applied policy research.

35. Sawhney A, Thacoor A, Akhavani M. Wide awake local anaesthetic no tourniquet (WALANT) technique in hand trauma surgery: A prospective study of efficacy and peri-operative patient experience. Journal of plastic, reconstructive & aesthetic surgery : JPRAS. 2021;74(9):2392-442.

For peer review only

Figure

Figure 1: Flow chart on patient's journey throughout the study

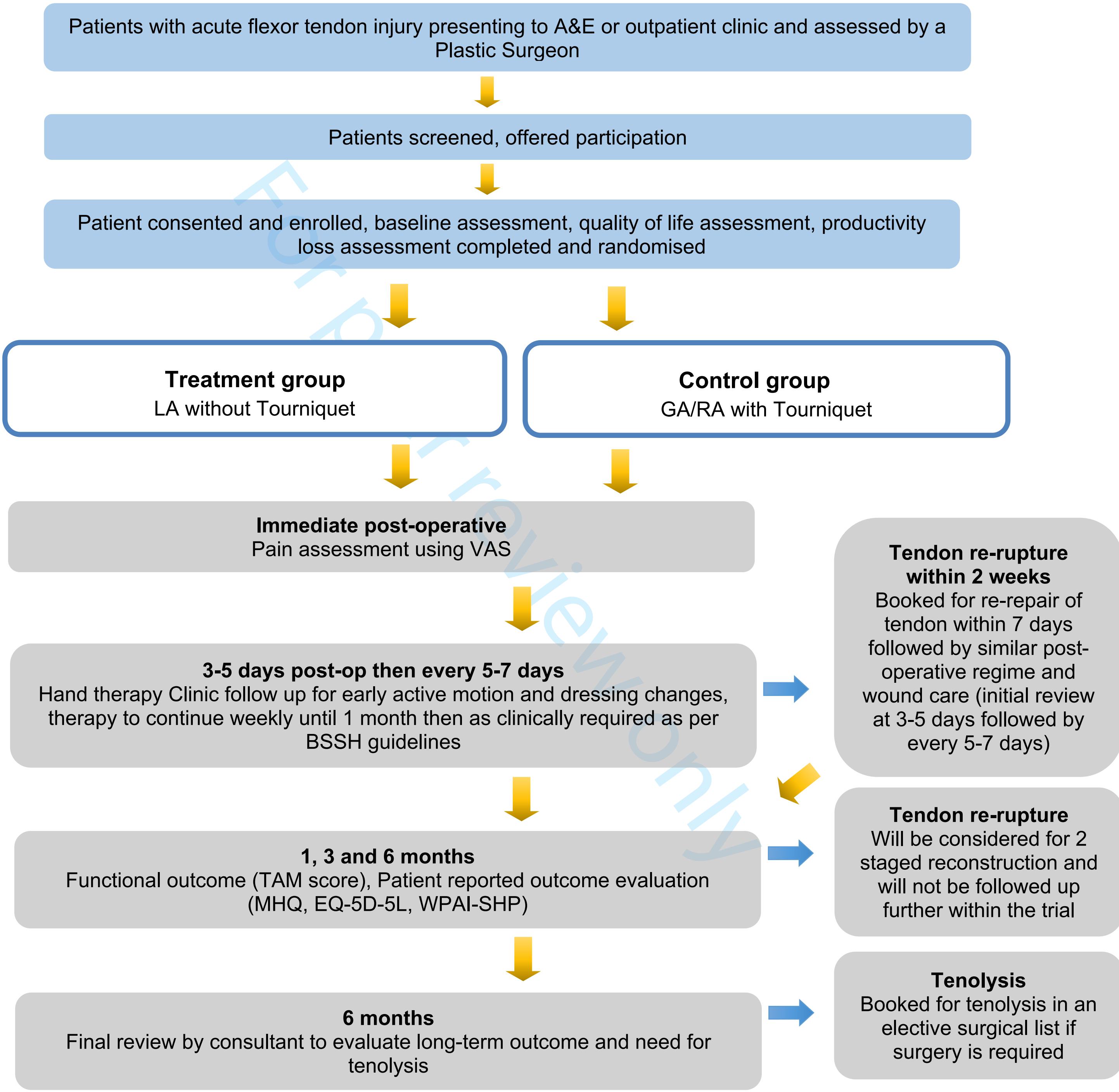

For peer review only

Author's contributions:

AM is the chief investigator for the trial. DN, JW, and WB are site PIs for the trial. MK, HMD, MP, CVP, NB, NW, JW, DN, AM are the grant holders and jointly contributed to the conception and design of the study. MK and RF drafted this manuscript. JB, HMD, MP, CVP, AP, NB, NW, WB, JW, AM, DN revised this manuscript. All authors read and approved the final manuscript.

Funding:

This project is funded by the National Institute for Health Research (NIHR) under its Research for Patient Benefit (RfPB) Programme (Grant Reference Number NIHR203610). The views expressed are those of the authors and not necessarily those of the NIHR or the Department of Health and Social Care.

Acknowledgements:

The authors would like to thank our patient representatives for their involvement in designing this study.

Conflicts of interest/financial disclosure:

The authors declare that the research was conducted in the absence of any commercial or financial relationships that could be construed as a potential conflict of interest.

For peer review only

Reporting checklist for protocol of a clinical trial.

Based on the SPIRIT guidelines.

Instructions to authors

Complete this checklist by entering the page numbers from your manuscript where readers will find each of the items listed below.

Your article may not currently address all the items on the checklist. Please modify your text to include the missing information. If you are certain that an item does not apply, please write "n/a" and provide a short explanation.

Upload your completed checklist as an extra file when you submit to a journal.

In your methods section, say that you used the SPIRITreporting guidelines, and cite them as:

Chan A-W, Tetzlaff JM, Gøtzsche PC, Altman DG, Mann H, Berlin J, Dickersin K, Hróbjartsson A, Schulz KF, Parulekar WR, Krleža-Jerić K, Laupacis A, Moher D. SPIRIT 2013 Explanation and Elaboration: Guidance for protocols of clinical trials. BMJ. 2013;346:e7586

		Reporting Item	Page Number
**Administrative information**			
Title	#1	Descriptive title identifying the study design, population, interventions, and, if applicable, trial acronym	1
Trial registration	#2a	Trial identifier and registry name. If not yet registered, name of intended registry	3
Trial registration: data set	#2b	All items from the World Health Organization Trial Registration Data Set	3
Protocol version	#3	Date and version identifier	2
Funding	#4	Sources and types of financial, material, and other support	23
Roles and responsibilities: contributorship	#5a	Names, affiliations, and roles of protocol contributors	23

For peer review only

Roles and responsibilities: sponsor contact information	#5b	Name and contact information for the trial sponsor	23
Roles and responsibilities: sponsor and funder	#5c	Role of study sponsor and funders, if any, in study design; collection, management, analysis, and interpretation of data; writing of the report; and the decision to submit the report for publication, including whether they will have ultimate authority over any of these activities	23
Roles and responsibilities: committees	#5d	Composition, roles, and responsibilities of the coordinating centre, steering committee, endpoint adjudication committee, data management team, and other individuals or groups overseeing the trial, if applicable (see Item 21a for data monitoring committee)	23
**Introduction**			
Background and rationale	#6a	Description of research question and justification for undertaking the trial, including summary of relevant studies (published and unpublished) examining benefits and harms for each intervention	5
Background and rationale: choice of comparators	#6b	Explanation for choice of comparators	6
Objectives	#7	Specific objectives or hypotheses	7
Trial design	#8	Description of trial design including type of trial (eg, parallel group, crossover, factorial, single group), allocation ratio, and framework (eg, superiority, equivalence, non-inferiority, exploratory)	7
**Methods: Participants, interventions, and outcomes**			
Study setting	#9	Description of study settings (eg, community clinic, academic hospital) and list of countries where data will be collected. Reference to where list of study sites can be obtained	7
Eligibility criteria	#10	Inclusion and exclusion criteria for participants. If applicable, eligibility criteria for study centres and individuals who will	7

For peer review only

		perform the interventions (eg, surgeons, psychotherapists)	
Interventions: description	#11a	Interventions for each group with sufficient detail to allow replication, including how and when they will be administered	8, 9
Interventions: modifications	#11b	Criteria for discontinuing or modifying allocated interventions for a given trial participant (eg, drug dose change in response to harms, participant request, or improving / worsening disease)	8 - 9
Interventions: adherance	#11c	Strategies to improve adherence to intervention protocols, and any procedures for monitoring adherence (eg, drug tablet return; laboratory tests)	8 - 9
Interventions: concomitant care	#11d	Relevant concomitant care and interventions that are permitted or prohibited during the trial	8 - 9
Outcomes	#12	Primary, secondary, and other outcomes, including the specific measurement variable (eg, systolic blood pressure), analysis metric (eg, change from baseline, final value, time to event), method of aggregation (eg, median, proportion), and time point for each outcome. Explanation of the clinical relevance of chosen efficacy and harm outcomes is strongly recommended	9 - 11
Participant timeline	#13	Time schedule of enrolment, interventions (including any run-ins and washouts), assessments, and visits for participants. A schematic diagram is highly recommended (see Figure)	11
Sample size	#14	Estimated number of participants needed to achieve study objectives and how it was determined, including clinical and statistical assumptions supporting any sample size calculations	11 - 12
Recruitment	#15	Strategies for achieving adequate participant enrolment to reach target sample size	11 - 12
**Methods: Assignment of interventions (for controlled trials)**			
Allocation: sequence generation	#16a	Method of generating the allocation sequence (eg, computer-generated random numbers), and list of any factors for stratification. To reduce predictability of a random sequence, details of any planned restriction (eg, blocking) should be provided in a separate document that is unavailable to those who enrol participants or assign interventions	12

For peer review only

Allocation concealment mechanism	#16b	Mechanism of implementing the allocation sequence (eg, central telephone; sequentially numbered, opaque, sealed envelopes), describing any steps to conceal the sequence until interventions are assigned	12
Allocation: implementation	#16c	Who will generate the allocation sequence, who will enrol participants, and who will assign participants to interventions	12
Blinding (masking)	#17a	Who will be blinded after assignment to interventions (eg, trial participants, care providers, outcome assessors, data analysts), and how	12
Blinding (masking): emergency unblinding	#17b	If blinded, circumstances under which unblinding is permissible, and procedure for revealing a participant's allocated intervention during the trial	12
**Methods: Data collection, management, and analysis**			
Data collection plan	#18a	Plans for assessment and collection of outcome, baseline, and other trial data, including any related processes to promote data quality (eg, duplicate measurements, training of assessors) and a description of study instruments (eg, questionnaires, laboratory tests) along with their reliability and validity, if known. Reference to where data collection forms can be found, if not in the protocol	12
Data collection plan: retention	#18b	Plans to promote participant retention and complete follow-up, including list of any outcome data to be collected for participants who discontinue or deviate from intervention protocols	12
Data management	#19	Plans for data entry, coding, security, and storage, including any related processes to promote data quality (eg, double data entry; range checks for data values). Reference to where details of data management procedures can be found, if not in the protocol	13
Statistics: outcomes	#20a	Statistical methods for analysing primary and secondary outcomes. Reference to where other details of the statistical analysis plan can be found, if not in the protocol	13 - 15
Statistics: additional analyses	#20b	Methods for any additional analyses (eg, subgroup and adjusted analyses)	13 - 15

For peer review only

Statistics: analysis population and missing data	#20c	Definition of analysis population relating to protocol non-adherence (eg, as randomised analysis), and any statistical methods to handle missing data (eg, multiple imputation)	13 - 15
**Methods: Monitoring**			
Data monitoring: formal committee	#21a	Composition of data monitoring committee (DMC); summary of its role and reporting structure; statement of whether it is independent from the sponsor and competing interests; and reference to where further details about its charter can be found, if not in the protocol. Alternatively, an explanation of why a DMC is not needed	12
Data monitoring: interim analysis	#21b	Description of any interim analyses and stopping guidelines, including who will have access to these interim results and make the final decision to terminate the trial	12
Harms	#22	Plans for collecting, assessing, reporting, and managing solicited and spontaneously reported adverse events and other unintended effects of trial interventions or trial conduct	12
Auditing	#23	Frequency and procedures for auditing trial conduct, if any, and whether the process will be independent from investigators and the sponsor	12
**Ethics and dissemination**			
Research ethics approval	#24	Plans for seeking research ethics committee / institutional review board (REC / IRB) approval	16 - 17
Protocol amendments	#25	Plans for communicating important protocol modifications (eg, changes to eligibility criteria, outcomes, analyses) to relevant parties (eg, investigators, REC / IRBs, trial participants, trial registries, journals, regulators)	16 - 17
Consent or assent	#26a	Who will obtain informed consent or assent from potential trial participants or authorised surrogates, and how (see Item 32)	11
Consent or assent: ancillary studies	#26b	Additional consent provisions for collection and use of participant data and biological specimens in ancillary studies, if applicable	11
Confidentiality	#27	How personal information about potential and enrolled participants will be collected, shared, and maintained in order to protect confidentiality before, during, and after the trial	13

For peer review only

Declaration of interests	#28	Financial and other competing interests for principal investigators for the overall trial and each study site	23
Data access	#29	Statement of who will have access to the final trial dataset, and disclosure of contractual agreements that limit such access for investigators	13, 23
Ancillary and post trial care	#30	Provisions, if any, for ancillary and post-trial care, and for compensation to those who suffer harm from trial participation	NA
Dissemination policy: trial results	#31a	Plans for investigators and sponsor to communicate trial results to participants, healthcare professionals, the public, and other relevant groups (eg, via publication, reporting in results databases, or other data sharing arrangements), including any publication restrictions	16 - 17
Dissemination policy: authorship	#31b	Authorship eligibility guidelines and any intended use of professional writers	NA
Dissemination policy: reproducible research	#31c	Plans, if any, for granting public access to the full protocol, participant-level dataset, and statistical code	NA
**Appendices**			
Informed consent materials	#32	Model consent form and other related documentation given to participants and authorised surrogates	NA
Biological specimens	#33	Plans for collection, laboratory evaluation, and storage of biological specimens for genetic or molecular analysis in the current trial and for future use in ancillary studies, if applicable	NA

The SPIRIT Explanation and Elaboration paper is distributed under the terms of the Creative Commons Attribution License CC-BY-NC. This checklist was completed on 25. April 2023 using https://www.goodreports.org/, a tool made by the EQUATOR Network in collaboration with Penelope.ai

For peer review only

BMJ Open

WAFER Trial: A study protocol for a feasibility randomised controlled trial comparing Wide-awake Local Anaesthesia No Tourniquet (WALANT) to General and Regional Anaesthesia with tourniquet for Flexor Tendon Repair

Journal:	*BMJ Open*
Manuscript ID	bmjopen-2023-075440.R1
Article Type:	Protocol
Date Submitted by the Author:	20-Jul-2023

Complete List of Authors:	Kanapathy, Muholan; University College London, Division of Surgery and Interventional Science ; Royal Free NHS Foundation Trust Hospital, Department of Plastic and Reconstructive Surgery<br>Faderani, Ryan; Royal Free NHS Foundation Trust Hospital, Department of Plastic and Reconstructive Surgery; University College London<br>Bray, Juliette; Royal Free NHS Foundation Trust Hospital, Department of Plastic and Reconstructive Surgery<br>Dehbi, Hakim-Moulay; University College London Institute of Clinical Trials and Methodology<br>Panca, Monica; University College London, Comprehensive Clinical Trials Unit<br>Vindrola-Padros, Cecilia; University College London, Department of Targeted Intervention<br>Prasad, Anjana; Royal Free NHS Foundation Trust Hospital, Department of Anaesthesia<br>Burr, Nicola; Royal Free NHS Foundation Trust Hospital, Department of Plastic and Reconstructive Surgery<br>Williams, Norman; University College London, UCL Division of Surgery & Interventional Science<br>Al-Ajam, Yazan; Royal Free NHS Foundation Trust Hospital, Department of Plastic and Reconstructive Surgery<br>Bhat, Waseem; Leeds Teaching Hospitals NHS Trust, Department of Plastic and Reconstructive Surgery<br>Wong, Jason ; The University of Manchester Faculty of Biology Medicine and Health, Blond McIndoe Laboratories, Division of Cell Matrix Biology and Regenerative Medicine<br>Mosahebi, Afshin; University College London Division of Surgery and Interventional Science; Royal Free NHS Foundation Trust Hospital, Department of Plastic and Reconstructive Surgery<br>Nikkhah, Dariush; Royal Free NHS Foundation Trust Hospital, Department of Plastic and Reconstructive Surgery; University College London

**Primary Subject Heading**:	Surgery
Secondary Subject Heading:	Anaesthesia, Emergency medicine

Keywords:	ANAESTHETICS, Anaesthesia in orthopaedics < ANAESTHETICS, Hand & wrist < ORTHOPAEDIC & TRAUMA SURGERY, PLASTIC & RECONSTRUCTIVE SURGERY, Plastic & reconstructive surgery < SURGERY

SCHOLARONE™
Manuscripts

Title Page

Title:

WAFER Trial: A study protocol for a feasibility randomised controlled trial comparing Wide-awake Local Anaesthesia No Tourniquet (WALANT) to General and Regional Anaesthesia with tourniquet for Flexor Tendon Repair

Authors:

Mr Muholan Kanapathy MD MRCS MSc PhD [1, 2]

Mr Ryan Faderani MBBS BSc MRCS FHEA [1, 3]

Miss Juliette Bray BSc (Hons) [1]

Dr Hakim-Moulay Dehbi PhD [4]

Miss Monica Panca MSc [4]

Prof Cecilia Vindrola-Padros BA MA PhD [5]

Dr Anjana Prasad BMedSci (Hons) BMBS FRCA [6]

Miss Nicola Burr Grad Dip Phys MSc [2]

Prof Norman Williams BSc PhD CSci MICR FIBMS [2]

Mr Yazan Al-Ajam MBChB FRCS (plast) [1]

Mr Waseem Bhat MD MA MSc FRCS, Dipl Hand Surg [7]

Mr Jason Wong MBChB FHEA PhD FRCS (plast) [8,9]

Prof Afshin Mosahebi MBBS MBA PhD FRCS (plast) [1,2] *

Mr Dariush Nikkhah BM MSc FRCS (plast) [1,2,3] *

* Co-senior Authors

Affiliations:

[1]Department of Plastic and Reconstructive Surgery, Royal Free NHS Foundation Trust Hospital, London, United Kingdom

[2] Division of Surgery & Interventional Science, University College London, United Kingdom

[3] Faculty of Medical sciences, University College London, United Kingdom

[4] Comprehensive Clinical Trials Unit, University College London, United Kingdom

For peer review only

[5] Department of Targeted Intervention, University College London, United Kingdom

[6] Department of Anaesthesia, Royal Free NHS Foundation Trust Hospital, London, United Kingdom

[7] Department of Plastic and Reconstructive Surgery, Leeds Teaching Hospitals NHS trust, Leeds, United Kingdom

[8] Department of Plastic and Reconstructive Surgery, Manchester University NHS Foundation Trust, Manchester, United Kingdom

[9] Blond McIndoe Laboratories, Division of Cell Matrix Biology and Regenerative Medicine, Manchester Academic Health Science Centre, School of Biological Sciences, Faculty of Biology, Medicine and Health, The University of Manchester, Manchester, United Kingdom

Corresponding author:

Mr Ryan Faderani

Department of Plastic and Reconstructive Surgery, Royal Free NHS Foundation Trust Hospital, London, United Kingdom

Email: Ryan.faderani.13@ucl.ac.uk

Protocol Version: 1.2 8/2/2023

Key words:

Tendon, Flexor tendon, Hand Surgery, Anaesthesia, WALANT

Word Count: 3871

For peer review only

Abstract

Introduction:

Flexor tendons are traditionally repaired under either general anaesthesia(GA) or regional anaesthesia(RA), allowing for the use of an arm tourniquet to minimise blood loss and establish a bloodless surgical field. However, the use of tourniquets exposes the patient to certain risks, including skin, muscle, and nerve injuries. A recent advancement in anaesthesia delivery involves the use of a wide-awake approach where no sedation nor tourniquets are used (WALANT). WALANT utilises local anaesthetic with adrenaline to provide pain relief and vasoconstriction, reducing operative bleeding. Several studies revealed potential benefits for WALANT compared to GA or RA. However, there remains a paucity of high-quality evidence to support the use of WALANT. As a result of this uncertainty, the clinical practice varies considerably. We aim to evaluate the feasibility of WALANT as an alternative to GA and RA in patients undergoing surgical repair of flexor tendon injuries. This involves addressing factors such as clinician and patient support for a trial, clinical equipoise, trial recruitment and dropout, and the most relevant outcomes measures for a future definitive trial.

Methods and analysis:

WAFER is a multi-centre, single-blinded, parallel group, randomised controlled trial (RCT) to assess the feasibility of WALANT versus RA and GA. The target population is patients with acute traumatic flexor tendon injuries, across three major hand surgery units in England involving a total of 60 participants. Outcome assessors will be blinded. The primary outcome will be the ability to recruit patients into the trial, whilst secondary outcomes include difference in functional outcome, patient-reported outcome measures, health-related quality of life, cost-effectiveness, and complication rates.

Ethics and dissemination:

Ethical approval was obtained from the London – City and East Research Ethics Committee (22/PR/1197). Findings will be disseminated through peer-reviewed publication, conferences, patient information websites and social media networks.

For peer review only

Trial registration:

ISRCTN identifier:15052559

Strengths and limitations of this study

- This study will use mixed methods which includes quantitative and qualitative measures to assess the feasibility of conducting a randomised controlled trial on performing flexor tendon repair under WALANT compared to general or regional anaesthesia with tourniquet.
- The inclusion of comprehensive outcome measures in the study will offer valuable insights into both the objective and patient-reported impact of flexor tendon injury.
- This study includes several major hand surgery units from different regions of the country to be inclusive and to identify differences and challenges faced at different units that are recruiting into the same trial to facilitate future study design.
- This study was designed and run by plastic surgical trainees alongside consultant plastic surgeons and surgical trials unit which emphasises the important role that surgical trainees play in surgical research
-
- One limitation of this study is its feasibility nature, which means that the sample size is not sufficient to detect differences between groups with a high degree of confidence.

For peer review only

Introduction

Hand injuries account for up to 20% of all presentations to emergency departments, equating to 4.6 million patients annually in the United Kingdom alone, and resulting in an average of 8 weeks of lost workdays (1, 2). In patients with hand injuries, tendon lacerations are common and are reported in 54.8% of patients with a shallow laceration and 92.5% with deeper injury (3). Patients with complete tendon laceration will require surgical repair, which is typically performed in the operating room, using an arm tourniquet to minimise blood loss and provide a clear surgical field (4). The use of a tourniquet is associated with significant discomfort and is not well tolerated by patients without general anaesthesia (GA) or regional anaesthesia (RA) (5).

A recent advancement in the method of anaesthesia delivery in hand surgery involves the introduction of Wide-awake Local Anaesthesia No Tourniquet (WALANT) (6,7,8). WALANT involves administering local anaesthesia (LA) with adrenaline for anaesthetic and vasoconstriction effect respectively (8). The vasoconstrictive effect of adrenaline achieves reduced bleeding at the site of surgery, hence acting as a chemical tourniquet and eliminating the need for a mechanical arm tourniquet. Removing the need for tourniquet and therefore GA/RA has several potential advantages for patients. Firstly, patients can make immediate post-operative recovery, ultimately shortening the patient stay in hospital. GA is associated with side effects such as nausea and vomiting along with serious cardiac and respiratory risks especially amongst patients undergoing emergency surgery, elderly, smokers, and overweight patients (9). RA avoids these risks but exposes the patient to the risk of permanent or temporary nerve damage (1 in 10) and failure of providing adequate pain relief resulting in conversion to GA (3 in 100) (10, 11). Tourniquet use has also been linked to increased post-operative pain and side effects such as muscle, nerve and skin injury, and post-tourniquet thrombosis (5, 12).

While WALANT requires multiple injections that may cause temporary discomfort, it avoids many of the significant issues associated with GA and RA. Furthermore, patients who undergo WALANT have been reported to have a decreased need for post-operative pain relief (13). Additionally, WALANT allows the surgeon to intraoperatively evaluate the tendon repair while the patient actively moves their fingers, allowing adjustments to be made before skin closure.

This has been shown to improve surgical outcomes and reduce the need for revision surgery (14, 15). Patients can also receive education during the procedure to aid their rehabilitation (7). There are also potential economic and environmental benefits to using WALANT, as it does not require an anaesthetist, requires fewer clinical staff (anaesthetist, anaesthetic assistant, recovery nurse), and produces fewer anaesthetic waste gases (16, 17).

Rationale

In recent years, WALANT has gained significant popularity as an alternative to GA or RA with tourniquet for flexor tendon repairs (18). While studies have suggested that it may have similar safety and potential benefits in comparison to GA or RA with a tourniquet, it should be noted that these studies are either retrospective in nature or lack sufficient statistical power to make definitive conclusions (19 – 22). Furthermore, it is important to determine the outcomes that are important to be measured, evaluate equipoise amongst clinician to recruit to a study comparing WALANT against GA/RA, and acceptability of the trial to patients and clinician before a definitive trial can be carried out. In addition, because of the uncertainty about the effectiveness and cost-effectiveness of WALANT compared to standard of care and the variation is clinical practice, a definitive RCT on the effectiveness and cost-effectiveness of WALANT is necessary. A feasibility study is hence necessary to help with the design of the definitive RCT. The WAFER trial therefore aims to evaluate the feasibility of WALANT as an alternative to GA or RA in patients undergoing surgical repair of traumatic laceration of flexor tendons of the hand.

For peer review only

Methods and analysis

Trial design

The WAFER trial is a multicentre, assessor-blinded, feasibility RCT with 2 parallel groups (WALANT vs. GA/RA) conducted in 3 sites within the UK. Eligible patients will be randomised to WALANT or GA/RA for flexor tendon repair using a computerised randomisation method. This protocol is reported in accordance with SPIRIT 2013 guidelines (23).

Patient and public involvement

Patient representatives who have had flexor tendon injury were involved in the design of this trial by providing feedback via online surveys and focus group meetings. A total of 27 patients were involved in the online survey and another 12 patients were involved in focus group meetings to identify priorities and outcomes of a flexor tendon study. Two patient representatives have been involved in designing and revising the trial to ensure that patient interests are central to the study design and that the trial is written clearly avoiding excessive or unnecessary medical jargon.

Study setting

Participants will be recruited at the Royal Free Hospital, Manchester University NHS Foundation Trust, and Leeds Teaching Hospital NHS Trust. This trial is open to recruiting additional sites through NIHR Clinical Research Network (CRN).

Eligibility criteria

Patients presenting to the Accident and Emergency (A&E) unit or outpatient clinics with acute traumatic flexor tendon ruptures will be reviewed by a hand surgeon and assessed. Normal clinical practice is that all patients will be assessed clinically and if the flexor tendon is lacerated, it will be scheduled for repair under GA/RA or WALANT in the operating theatre. Upon scheduling the patient for surgery, patients will be flagged to the research team. At this time, patients will be screened and where appropriate be offered a patient information sheet for inclusion into the trial. This process will include explanation of the aims, methods of

For peer review only - http://bmjopen.bmj.com/site/about/guidelines.xhtml

anaesthesia and surgery, anticipated benefits, and potential hazards of the study. Patients will be given sufficient time (24 hours or more if needed) to consider whether they wish to participate. Once the patients are ready for surgery, following review by the study team, patients will be assessed clinically to confirm 'inclusion and exclusion criteria' are met and informed consent form will be completed, following which patients will be randomised to either of the treatment arm and enrolled into the study. Patient related data will be recorded in a Patient Assessment Form.

Inclusion criteria

1. Adults ≥ 18 years old.
2. Clinical diagnosis of acute flexor tendon injury within 2 weeks of injury (≤2 weeks from injury to date of surgery).
3. Complete laceration (100% transection) of Flexor digitorum profundus, and/or Flexor digitorum superficialis, and/or Flexor pollicis longus of the hand.
4. Single digit or two digits injury involving flexor zone 1 to 3.
5. Patient understands and is willing to participate and can comply with the follow-up regime.

Exclusion criteria

1. Tendon not amendable to primary repair (gross wound contamination, segmental tendon loss, associated fractures, or mangled hand injuries).
2. Secondary tendon repair or reconstruction.
3. History of allergy to LA.
4. Refusal to have LA or deemed non-cooperative to be performed without sedation.
5. Pre-existing deformity of finger or hand.
6. High risk for GA or not fit for surgery (ASA ≥ 4).

Interventions

WALANT

For peer review only

Patients will have the procedure in an operating theatre under WALANT without an arm tourniquet. WALANT uses a mixture of local anaesthesia with adrenaline to provide anaesthesia and haemostasis. A mixture of 1% xylocaine with adrenaline 1:200,000 will be injected into the digit or hand requiring surgery. This mixture is routinely used in clinical practice (24, 25). The mixture is used in a "field block" technique where 2ml is injected in the subcutaneous fat in the middle of the base of the proximal and middle phalanges between both digital nerves, and 1 ml is injected in the distal phalanx. In addition, 10- 15ml local anaesthesia will be injected in the palm where dissection will be performed (26).

Following injection, surgeons typically wait about 20 minutes for the haemostatic effect of adrenaline before incision. The flexor tendon repair will be performed as per standard practice using 4-strand core repair with epitendinous stitch as per the British Society for Surgery of the Hand (BSSH) guidelines (27).

General / Regional anaesthesia

Patients will have the procedure in an operating theatre under either RA or GA as per the anaesthetist's safe standard practice. RA is preferentially chosen unless there is a contra-indication. An axillary, supraclavicular or infraclavicular brachial plexus block is performed awake using ultrasound guidance and may be supplemented with ultrasound guided peripheral nerve (ulnar and/or median) blocks. Lignocaine (1% or 2%) and/or Bupivacaine (0.25% or 0.5%) with or without 1:200,000 adrenaline is given for the blocks at a calculated safe dose depending on the patient's weight. GA is administered either as inhalational anaesthesia using sevoflurane or total intravenous anaesthesia with propofol and a short-acting opiate (remifentanil or alfentanil). Analgesia is given intravenously as per the World Health Organisation analgesic ladder during a GA, with LA topical infiltration by the surgeons. The surgical procedure will be performed as per standard practice with the use of an arm tourniquet for haemostasis. The tendon repair will be performed with 4-strand core repair with epitendinous stitch as per BSSH guidelines (27).

Post-operative care

All patients will have a hand therapy appointment within 3-5 days of the operation to commence early active motion and have dressings changed (as per the British Society for

For peer review only

Surgery of the Hand guidelines) (27). All patients will have follow-up appointments with the hand therapy team for ongoing rehabilitation and dressing changes every 5-7 days until the wound heals. They will then have follow-up appointments every 5-7 days for 1 month as clinically required.

Study outcomes:

Primary outcome & measures

The primary outcome of the trial will be the ability to recruit patients at the selected sites (recruitment rate). Feasibility will be monitored through screening and randomisation logs with the aim to recruit 1 to 2 participants per month per site. Advisory group meetings involving patients, clinicians and methodologist will explore the following:

- equipoise and willingness of clinicians to recruit and patients to participate.
- withdrawals from the trial or failure to complete the follow-up visits.
- the optimal primary outcome for subsequent definitive trial on completion of the trial and presentation of the data (based on clinical and qualitative data).

The primary outcome measures are:

1. Recruitment into a trial of this nature (recruitment rate at each centre over 16 months)
2. Support for the trial from involved clinicians (Assessed through qualitative interviews)
3. Equipoise amongst clinicians and patients (Assessed through qualitative interviews)
4. Rate of withdrawal/drop-out from the study (Assessed throughout the trial)

Secondary outcome & measures

Our secondary outcome is to determine the primary outcome and outcome measures for a subsequent definitive randomised controlled trial. Several secondary outcome measures will be collected in order to help with the design of a definitive trial to determine the effectiveness and cost-effectiveness of performing flexor tendon repair under WALANT vs RA/GA. The secondary outcome measures are:

For peer review only

1. Difference in the proportion of good functional outcome measured using Total Active Motion (TAM) score at 1, 3 and 6 months. The TAM score measures the degree of movements of the finger joints using a goniometer, an instrument which measures an angle, and compares it against the uninjured contralateral finger which gives percentage of normal. Results more than 75% is classified as good functional outcome.
2. Difference in grip strength and pinch strength at 1, 3, and 6 months.
3. Difference in post-operative finger oedema – measured at 1, 3, and 6 months.
4. Patient reported outcome at 1, 3 and 6 months using the Michigan Hand Outcomes Questionnaire (MHQ) (patient-reported questionnaire on interference with daily life measured on a 5-point Likert scale involving 6 domains). (28)
5. Productivity impact and health-related quality of life-assessed at baseline, 1, 3 and 6 months using the Work Productivity and Activity Impairment Questionnaire: Specific Health Problem (WPAI-SHP) and EQ-5D-5L (evaluates 5 main health domains in 5 levels of severity) questionnaires. (29, 30)
6. Pain-assessment using Visual analogue score (VAS) tool, which uses a 10-point Likert scale to assess pain immediately post-surgery.
7. Difference in proportion of patients with tendon ruptures following repair at 1, 3 and 6 months.
8. Difference in proportion of patients with tenolysis at 6 months.
9. Difference in proportion of patients with overall complications at 6 months.
10. Adverse events.
11. Healthcare resource use.

Participant timeline

The study was opened for recruitment in March 2023 and is anticipated to close in September 2024. Each patient will be followed up for 6 months. Figure 1 summarises patient's journey throughout the trial.

For peer review only

Sample size

A sample size of 200 eligible patients will be approached and asked to give their consent to be randomised into the trial. We anticipate that the approximate recruitment (randomisation) rate will be 30% of the approached patients. As such, 200 patients would be sufficient to be able to estimate the consent rate to within ± 0.07 through construction of 95% confidence interval for the recruitment rate. Under the assumption that the recruitment rate is approximately 30%, we anticipate that up to 60 patients will be randomised (1:1 WALANT:RA/GA) to take part in the study. Our local audit informed that on average over 100 cases of tendon repairs are performed per year by each centre of varying complexity (31).

Randomisation

Patients who meet the eligibility criteria will be randomised to have flexor tendon repair under GA/RA or WALANT on a 1:1 ratio. Randomisation will be performed by a study researcher using REDCap. Participants will be deemed enrolled into the study once they have been randomised to either of the study arms.

Blinding

The surgical team, clinical staff, and patient will not be blinded to the randomised intervention status. The hand therapists assessing the outcomes during follow-up visits will be blinded to the allocation.

Data collection and management

Data will be collected at the initial assessment, intra-operatively and at follow-up assessments. Data collection is completed by trained surgeons and hand therapists using paper 'clinical research form '(CRF). A local data manager at each site will then enter the data from the CRF's onto REDCap electronic data tools database. Completeness of data will be ensured by reviewing the patient's medical notes. A central data manager will ensure the accuracy of the data collection by preforming sample assessments at regular intervals. Any adverse events will be recorded and reported to the primary investigators and institutional ethics committee.

For peer review only

Qualitative assessments

We will conduct semi-structured interviews informed by a topic guide developed from the existing literature in conjunction with the trial management group, which includes patient representatives. We will explore the reasons for participation and non-participation of patients and the acceptability of the trial to clinicians and patients to assist in improvement of recruitment strategies employed for the definitive trial. The interview will be audio recorded, but the researcher will also take notes. These notes will be summarised and added to a RREAL Sheet per study site so emerging findings can be shared with the trial team as the qualitative evaluation is ongoing (32,33).

Participants will be divided into two groups according to whether they agreed (including cases of withdrawal from the trial) or did not agree to be randomised to the trial. The interviews will explore patient's perspectives of the treatment, their understanding of the two treatments, their reasons for taking part or refusing the trial, and their acceptability of randomisation between the procedures. Purposive sampling will be used to select participants according to their age, pre-existing comorbidities, and recruitment centre. A total of 40 patient interviews and 15 staff interviews will be conducted across the 3 sites. This method of sampling will ensure that a wide range of experiences and perceptions are collected. Interviews will continue until data saturation is reached. We will recruit patients who refused randomisation, those who consented, and those who withdrew consent. We will also recruit members of staff in charge of delivering the trial to understand trial delivery processes and identify any issues with recruitment. Recruitment to the interviews will take place alongside recruitment to the trial.

Data storage

The data extracted for the purposes of this study will be anonymised. All handling, processing, and storage of personal identifiable data and study data will be in accordance with the Data Protection Act 1998 and the NHS Code of Confidentiality.

For peer review only

Statistical analysis

Quantitative analysis:

The baseline characteristics of the two groups will be reported using means and standard deviations or medians and inter-quartile ranges for continuous variables, as appropriate, and frequency counts and percentages for categorical variables. The proportion of patients who consent to be randomised will be presented with a 95% confidence interval. For other outcomes, we will explore the difference in proportion (for binary outcomes) and mean or median difference (for continuous outcomes) between the two groups will be presented with associated 95% confidence intervals. No formal comparisons between the groups will be made and no hypothesis tests will be carried out. The results will inform us how sensitive the outcome measures are and, along with other information, will be used to determine the primary outcome of a subsequent large RCT. The results will also inform a sample size calculation for the primary outcome chosen.

Qualitative analysis:

Interviews will be transcribed verbatim and coded using NVivo Computer Assisted Qualitative Data Analysis Software. The transcripts will then be analysed using framework analysis (34). The transcripts will be analysed by two researchers. The first researcher will fully code and analyse all transcripts. A sample of these will then be analysed by a second researcher. The main themes to include in the framework will be double-checked by the second researcher, and any disagreements will be discussed and resolved within the research team. Information gained from the qualitative process evaluation will be used to inform the development of the protocol for the definitive trial. This may mean the development of training materials for staff, changes to participant information sheets, changes to recruitment processes, etc.

Health economic analysis:

This feasibility study will be used to plan the evaluation within the subsequent trial, which will aim to estimate the cost and the potential benefit in economic terms from the NHS perspective and personal social services. We will also explore the feasibility of collecting data to assess the costs for patients and families from a broader (societal) perspective.

Within the feasibility study of economic evaluation, we will:

1. Perform a literature search to identify economic studies and evidence available on similar interventions (e.g. to capture cost-effectiveness models and data that might be needed in addition to those collected within the trial).
2. Identify, in consultation with clinicians, the main cost components of WALANT, GA and RA (equipment, materials, staff cost).
3. Identify the resource use and unit cost data for each of these components.
4. Identify the potential costs of treating side effects in both treatments.
5. Identify potential instruments to estimate patient reported outcome and quality of life in patients in addition to MHQ and EQ-5D-5L questionnaires.
6. Identify the main costs for patients and families (e.g. transport costs, care givers ' costs, productivity losses) and best tools to collect the data.
7. Design a Markov model using data from the feasibility study to describe the patients pathway in the two treatment arms and to model costs and effects beyond-trial, assuming a life-time horizon (the model data will be chosen from up-to-date UK sources, published literature and clinicians and patient and public representatives

With the available data we will perform a preliminary analysis of the cost-utility of WALANT against RA/GA for flexor tendon repair to inform the future trial.

Discussion

The purpose of this study is to determine the feasibility of conducting a randomised controlled trial that compares the use of WALANT and RA/GA as methods of anaesthesia for flexor tendon repairs. The results of this study will inform the design of a larger, more comprehensive trial. By examining factors such as patient and clinician acceptability, recruitment rates, and key clinical outcome measures, the study aims to identify the most important and relevant considerations for a definitive trial. Additionally, the study will also evaluate the cost-effectiveness of WALANT as compared to the traditional standard of care in order to provide insights into the financial implications of using this method of anaesthesia.

For peer review only

In designing this study, we conducted a retrospective observational study, an online survey and performed a focus group meeting with experts in the field, including hand surgeons, hand therapists, and patients. In our retrospective study, we examined 151 patients involving a total of 251 tendon ruptures repaired either under WALANT or Regional/GA anesthesia (35). Our analysis revealed no significant differences in rates of tendon rupture, adhesions, infection, or hand function between the two groups. These findings suggest that WALANT may be a safe and effective alternative to GA/RA with comparable surgical and functional outcomes. Additionally, WALANT allowed for intraoperative testing of the repair, provided patient education, and had the potential to increase theatre efficiency and cost-effectiveness. However, this data is limited by the retrospective nature of the study, small sample size and potential confounders related to severity of injury, therefore the true difference between groups is not known. Meanwhile, an online survey involving 60 participants (26 hand surgeons, 7 hand therapists and 27 patients) followed by a focus group meeting involving 22 participants (8 hand surgeons, 2 hand therapist and 12 patients) were carried out to identify priorities and outcomes of a flexor tendon study. Most of our survey participants (95.3%) agreed that a trial comparing WALANT against RA/GA should be performed. 83.9% of clinicians were willing to recruit or encourage recruitment, while 83% of patients were willing to participate in such a study. The focus group participants agreed that the important outcomes that should be compared are functional outcome, patient satisfaction, and the difference in complications. The patients in our focus group mentioned that they would consider the new approach only if it provided at least equivalent hand function as the current standard of care. Our survey and focus group indicated good support from clinicians and patients to participate in a trial of this nature, however there is a need to evaluate equipoise amongst all parties. Our focus group also suggested that some outcomes are more important than others. Therefore, it is important to find out the acceptability of the trial to patients and clinicians, recruitment rate, primary outcome of subsequent definitive trial, and the difference in the primary outcome that can be considered a meaningful difference. Therefore, a feasibility study needs to be conducted to assess the practicality of conducting a larger, more conclusive trial, as well as to determine the most important outcomes to measure. This feasibility study will also provide valuable insights for future research in hand surgery, particularly with regards to study design and outcome selection.

For peer review only

Ethics and dissemination

This trial has approval from the London – City and East Research Ethics Committee (project ID: 22/PR/1197). This trial will be conducted in accordance with the Declaration of Helsinki and the recommendations of Good Clinical Practice. The trial has been registered in ISRCTN registry (ISRCTN identifier:15052559).

The results of the study will be reported and disseminated in peer-reviewed scientific journals, conference presentations, and website/online publications, as well as in internal reports. Reporting will be based on CONSORT guideline for reporting randomised trials. All publications will be forwarded to participants.

Trial status

This trial has begun recruitment in March 2023.

For peer review only

References

1. Wong JY. Time off work in hand injury patients. The Journal of hand surgery. 2008;33(5):718-25.

2. BSSH. Hand Surgery in the UK. Report of a Working Party. 2017. Available at: https://www.bssh.ac.uk/professionals/hand_surgery_in_the_uk.aspx. Accessed on: 25/10/2021.

3. Tuncali D, Yavuz N, Terzioglu A, Aslan G. The rate of upper-extremity deep-structure injuries through small penetrating lacerations. Annals of plastic surgery. 2005;55(2):146-8.

4. Gibson PD, Sobol GL, Ahmed IH. Zone II Flexor Tendon Repairs in the United States: Trends in Current Management. The Journal of hand surgery. 2017;42(2):e99-e108.

5. Noordin S, McEwen JA, Kragh JF, Jr., Eisen A, Masri BA. Surgical tourniquets in orthopaedics. The Journal of bone and joint surgery American volume. 2009;91(12):2958-67.

6. Nikkhah D, Ruston J, Pearl R, Blair J. 'WALANT carpal tunnel release: Technical considerations and pain outcomes'. Journal of plastic, reconstructive & aesthetic surgery : JPRAS. 2019;72(9):1576-606.

7. Lalonde D, Bell M, Benoit P, Sparkes G, Denkler K, Chang P. A multicenter prospective study of 3,110 consecutive cases of elective epinephrine use in the fingers and hand: the Dalhousie Project clinical phase. The Journal of hand surgery. 2005;30(5):1061-7.

8. Lalonde DH, Wong A. Dosage of local anesthesia in wide awake hand surgery. The Journal of hand surgery. 2013;38(10):2025-8.

9. National Health Service. Anaesthesia. Available at: https://www.nhs.uk/conditions/anaesthesia/. Accessed on 25/10/2021. NHS Evidence, 2018

10. Jeng CL, Torrillo TM, Rosenblatt MA. Complications of peripheral nerve blocks. British journal of anaesthesia. 2010;105 Suppl 1:i97-107.

11. Teunkens A, Vermeulen K, Belmans A, Degreef I, Van de Velde M, Rex S. Patient satisfaction with intravenous regional anaesthesia or an axillary block for minor ambulatory hand surgery: A randomised controlled study. European journal of anaesthesiology. 2020;37(10):847-56.

12. Gunasagaran J, Sean ES, Shivdas S, Amir S, Ahmad TS. Perceived comfort during minor hand surgeries with wide awake local anaesthesia no tourniquet (WALANT) versus local

For peer review only

anaesthesia (LA)/tourniquet. Journal of orthopaedic surgery (Hong Kong). 2017;25(3):2309499017739499.

13. Lalchandani GR, Halvorson RT, Rahgozar P, Immerman I. Wide-Awake Local Anesthesia for Minor Hand Surgery Associated With Lower Opioid Prescriptions, Morbidity, and Costs: A Nationwide Database Study. Journal of Hand Surgery Global Online. 2020;2(1):7-12.

14. Higgins A, Lalonde DH, Bell M, McKee D, Lalonde JF. Avoiding flexor tendon repair rupture with intraoperative total active movement examination. Plastic and reconstructive surgery. 2010;126(3):941-5

15. Kadhum, M., Georgiou, A., Kanapathy, M., Reissis, D., Akhavani, M., Burr, N., & Nikkhah, D. (2022). Operative outcomes for wide awake local anesthesia versus regional and general anesthesia for flexor tendon repair. Hand surgery & rehabilitation, 41(1), 125–130. https://doi.org/10.1016/j.hansur.2021.10.312

16. Yen CY, Ma CH, Wu CH, Yang SC, Jou IM, Tu YK. A cost and efficacy analysis of performing arthroscopic excision of wrist ganglions under wide-awake anaesthesia versus general anaesthesia. BMC musculoskeletal disorders. 2020;21(1):459.

17. Chatterjee A, McCarthy JE, Montagne SA, Leong K, Kerrigan CL. A cost, profit, and efficiency analysis of performing carpal tunnel surgery in the operating room versus the clinic setting in the United States. Annals of plastic surgery. 2011;66(3):245-8.

18. Søreide K, Hallet J, Matthews JB, Schnitzbauer AA, Line PD, Lai PBS, et al. Immediate and long-term impact of the COVID-19 pandemic on delivery of surgical services. The British journal of surgery. 2020

19. Reissis D, Georgiou A, Nikkhah D, O'Toole G. U.K. Response to the COVID-19 Pandemic: Managing Plastic Surgery Patients Safely. Plastic and reconstructive surgery. 2020;146(2):250e-1e.

20. Hobday D, Welman T, O'Neill N, Pahal GS. A protocol for wide awake local anaesthetic no tourniquet (WALANT) hand surgery in the context of the coronavirus disease 2019 (COVID-19) pandemic. The surgeon : journal of the Royal Colleges of Surgeons of Edinburgh and Ireland. 2020.

21. Nolan GS, Kiely AL, Madura T, Karantana A. Wide-awake local anaesthesia no tourniquet (WALANT) vs regional or general anaesthesia for flexor tendon repair in adults: protocol for a systematic review and meta-analysis. Systematic reviews. 2020;9(1):264.

For peer review only

22. Choukairi F, Ibrahim I, R NAM, Reid AJ, Winterton RI, Bedford JD, et al. Development of the Manchester wide-awake hand trauma service in 2020: the patient experience. The Journal of hand surgery, European volume. 2021;46(5):569-73.
23. Chan A-W, Tetzlaff JM, Altman DG, Dickersin K, Moher D. SPIRIT 2013: new guidance for content of clinical trial protocols. Lancet. 2013;381(9861):91–2.
24. Grandizio LC, Graham J, Klena JC. Current Trends in WALANT Surgery: A Survey of American Society for Surgery of the Hand Members. Journal of Hand Surgery Global Online. 2020;2(4):186-90.
25. Zargaran D, Zargaran A, Nikkhah D, Mosahebi A. WALANT Protocol: Stop before you block. Journal of plastic, reconstructive & aesthetic surgery : JPRAS. 2021;74(10):2776-820. (5)
26. Lalonde, D. H., & Martin, A. L. (2013). Wide-awake flexor tendon repair and early tendon mobilization in zones 1 and 2. Hand clinics, 29(2), 207–213. https://doi.org/10.1016/j.hcl.2013.02.009
27. The British Society for Surgery of the Hand, Lacerations with flexor tendon involvement guildelines, BSSH Standards of Care in Hand Trauma, https://www.bssh.ac.uk/bssh_standards_of_care_in_hand_trauma_.aspx 2020
28. Chung KC, Pillsbury MS, Walters MR, Hayward RA. Reliability and validity testing of the Michigan Hand Outcomes Questionnaire. J Hand Surg Am. 1998;23(4):575-87.
29. Reilly MC, Zbrozek AS, Dukes E: The validity and reproducibility of a work productivity and activity impairment measure. PharmacoEconomics 1993; 4(5):353-365.
30. Herdman M, Gudex C, Lloyd A, et al. Development and preliminary testing of the new five-level version of EQ-5D (EQ-5D-5L). Qual Life Res. 2011;20(10):1727-1736. doi:10.1007/s11136-011-9903-x
31. Faderani, R., Kanapathy, M., & Mosahebi, A. (2021). Hand injury: investigating the accuracy of referrals to a specialist trauma centre. Emergency nurse : the journal of the RCN Accident and Emergency Nursing Association, 29(3), 29–33. https://doi.org/10.7748/en.2021.e2065
32. Vindrola-Padros, C., Chisnall, G., Polanco, N., & San Juan, N. V. (2022). Iterative cycles in qualitative research: introducing the RREAL sheet as an innovative process. https://papers.ssrn.com/sol3/papers.cfm?abstract_id=4162797

For peer review only

33. Vindrola-Padros, C., Chisnall, G., Cooper, S., Dowrick, A., Djellouli, N., Symmons, S. M., ... & Johnson, G. A. (2020). Carrying out rapid qualitative research during a pandemic: emerging lessons from COVID-19. Qualitative health research, 30(14), 2192-2204.

34. Srivastava, A., & Thomson, S. B. (2009). Framework analysis: a qualitative methodology for applied policy research.

35. Sawhney A, Thacoor A, Akhavani M. Wide awake local anaesthetic no tourniquet (WALANT) technique in hand trauma surgery: A prospective study of efficacy and peri-operative patient experience. Journal of plastic, reconstructive & aesthetic surgery : JPRAS. 2021;74(9):2392-442.

For peer review only

Figure

Figure 1: Flow chart on patient’s journey throughout the study

For peer review only

Author's contributions:

AM is the chief investigator for the trial. DN, JW, and WB are site PIs for the trial. MK, HMD, MP, CVP, NB, NW, JW, DN, AM are the grant holders and jointly contributed to the conception and design of the study. MK and RF drafted this manuscript. JB, HMD, MP, CVP, AP, NB, NW, YA, WB, JW, AM, DN revised this manuscript. All authors read and approved the final manuscript.

Funding:

This project is funded by the National Institute for Health Research (NIHR) under its Research for Patient Benefit (RfPB) Programme (Grant Reference Number NIHR203610). The views expressed are those of the authors and not necessarily those of the NIHR or the Department of Health and Social Care.

Acknowledgements:

The authors would like to thank our patient representatives for their involvement in designing this study.

Conflicts of interest/financial disclosure:

The authors declare that the research was conducted in the absence of any commercial or financial relationships that could be construed as a potential conflict of interest.

For peer review only

Figure

Figure 1: Flow chart on patient's journey throughout the study

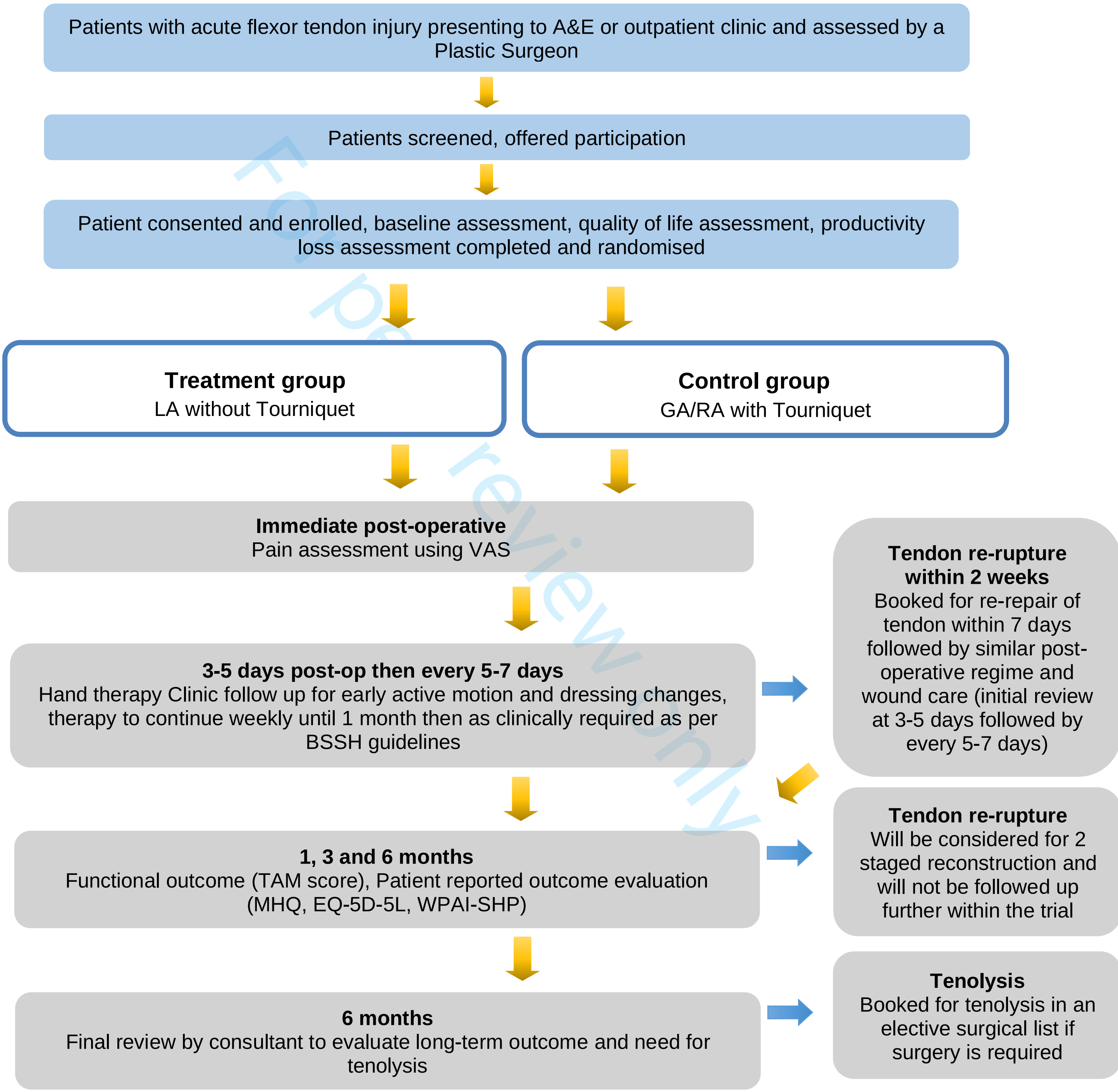

For peer review only

Reporting checklist for protocol of a clinical trial.

Based on the SPIRIT guidelines.

Instructions to authors

Complete this checklist by entering the page numbers from your manuscript where readers will find each of the items listed below.

Your article may not currently address all the items on the checklist. Please modify your text to include the missing information. If you are certain that an item does not apply, please write "n/a" and provide a short explanation.

Upload your completed checklist as an extra file when you submit to a journal.

In your methods section, say that you used the SPIRITreporting guidelines, and cite them as:

Chan A-W, Tetzlaff JM, Gøtzsche PC, Altman DG, Mann H, Berlin J, Dickersin K, Hróbjartsson A, Schulz KF, Parulekar WR, Krleža-Jerić K, Laupacis A, Moher D. SPIRIT 2013 Explanation and Elaboration: Guidance for protocols of clinical trials. BMJ. 2013;346:e7586

For peer review only

		Reporting Item	Page Number
**Administrative information**			
Title	#1	Descriptive title identifying the study design, population, interventions, and, if applicable, trial acronym	1
Trial registration	#2a	Trial identifier and registry name. If not yet registered, name of intended registry	3
Trial registration: data set	#2b	All items from the World Health Organization Trial Registration Data Set	3
Protocol version	#3	Date and version identifier	2
Funding	#4	Sources and types of financial, material, and other support	23
Roles and responsibilities: contributorship	#5a	Names, affiliations, and roles of protocol contributors	23

Roles and responsibilities: sponsor contact information	#5b	Name and contact information for the trial sponsor	23
Roles and responsibilities: sponsor and funder	#5c	Role of study sponsor and funders, if any, in study design; collection, management, analysis, and interpretation of data; writing of the report; and the decision to submit the report for publication, including whether they will have ultimate authority over any of these activities	23
Roles and responsibilities: committees	#5d	Composition, roles, and responsibilities of the coordinating centre, steering committee, endpoint adjudication committee, data management team, and other individuals or groups overseeing the trial, if applicable (see Item 21a for data monitoring committee)	23
**Introduction**			
Background and rationale	#6a	Description of research question and justification for undertaking the trial, including summary of relevant studies (published and unpublished) examining benefits and harms for each intervention	5
Background and rationale: choice of comparators	#6b	Explanation for choice of comparators	6
Objectives	#7	Specific objectives or hypotheses	7
Trial design	#8	Description of trial design including type of trial (eg, parallel group, crossover, factorial, single group), allocation ratio, and framework (eg, superiority, equivalence, non-inferiority, exploratory)	7
**Methods: Participants, interventions, and outcomes**			
Study setting	#9	Description of study settings (eg, community clinic, academic hospital) and list of countries where data will be collected. Reference to where list of study sites can be obtained	7
Eligibility criteria	#10	Inclusion and exclusion criteria for participants. If applicable, eligibility criteria for study centres and individuals who will	7

For peer review only

		perform the interventions (eg, surgeons, psychotherapists)	
Interventions: description	#11a	Interventions for each group with sufficient detail to allow replication, including how and when they will be administered	8, 9
Interventions: modifications	#11b	Criteria for discontinuing or modifying allocated interventions for a given trial participant (eg, drug dose change in response to harms, participant request, or improving / worsening disease)	8 - 9
Interventions: adherance	#11c	Strategies to improve adherence to intervention protocols, and any procedures for monitoring adherence (eg, drug tablet return; laboratory tests)	8 - 9
Interventions: concomitant care	#11d	Relevant concomitant care and interventions that are permitted or prohibited during the trial	8 - 9
Outcomes	#12	Primary, secondary, and other outcomes, including the specific measurement variable (eg, systolic blood pressure), analysis metric (eg, change from baseline, final value, time to event), method of aggregation (eg, median, proportion), and time point for each outcome. Explanation of the clinical relevance of chosen efficacy and harm outcomes is strongly recommended	9 - 11
Participant timeline	#13	Time schedule of enrolment, interventions (including any run-ins and washouts), assessments, and visits for participants. A schematic diagram is highly recommended (see Figure)	11
Sample size	#14	Estimated number of participants needed to achieve study objectives and how it was determined, including clinical and statistical assumptions supporting any sample size calculations	11 - 12
Recruitment	#15	Strategies for achieving adequate participant enrolment to reach target sample size	11 - 12
**Methods: Assignment of interventions (for controlled trials)**			
Allocation: sequence generation	#16a	Method of generating the allocation sequence (eg, computer-generated random numbers), and list of any factors for stratification. To reduce predictability of a random sequence, details of any planned restriction (eg, blocking) should be provided in a separate document that is unavailable to those who enrol participants or assign interventions	12

For peer review only

Allocation concealment mechanism	#16b	Mechanism of implementing the allocation sequence (eg, central telephone; sequentially numbered, opaque, sealed envelopes), describing any steps to conceal the sequence until interventions are assigned	12
Allocation: implementation	#16c	Who will generate the allocation sequence, who will enrol participants, and who will assign participants to interventions	12
Blinding (masking)	#17a	Who will be blinded after assignment to interventions (eg, trial participants, care providers, outcome assessors, data analysts), and how	12
Blinding (masking): emergency unblinding	#17b	If blinded, circumstances under which unblinding is permissible, and procedure for revealing a participant's allocated intervention during the trial	12
**Methods: Data collection, management, and analysis**			
Data collection plan	#18a	Plans for assessment and collection of outcome, baseline, and other trial data, including any related processes to promote data quality (eg, duplicate measurements, training of assessors) and a description of study instruments (eg, questionnaires, laboratory tests) along with their reliability and validity, if known. Reference to where data collection forms can be found, if not in the protocol	12
Data collection plan: retention	#18b	Plans to promote participant retention and complete follow-up, including list of any outcome data to be collected for participants who discontinue or deviate from intervention protocols	12
Data management	#19	Plans for data entry, coding, security, and storage, including any related processes to promote data quality (eg, double data entry; range checks for data values). Reference to where details of data management procedures can be found, if not in the protocol	13
Statistics: outcomes	#20a	Statistical methods for analysing primary and secondary outcomes. Reference to where other details of the statistical analysis plan can be found, if not in the protocol	13 - 15
Statistics: additional analyses	#20b	Methods for any additional analyses (eg, subgroup and adjusted analyses)	13 - 15

For peer review only

Statistics: analysis population and missing data	#20c	Definition of analysis population relating to protocol non-adherence (eg, as randomised analysis), and any statistical methods to handle missing data (eg, multiple imputation)	13 - 15
**Methods: Monitoring**			
Data monitoring: formal committee	#21a	Composition of data monitoring committee (DMC); summary of its role and reporting structure; statement of whether it is independent from the sponsor and competing interests; and reference to where further details about its charter can be found, if not in the protocol. Alternatively, an explanation of why a DMC is not needed	12
Data monitoring: interim analysis	#21b	Description of any interim analyses and stopping guidelines, including who will have access to these interim results and make the final decision to terminate the trial	12
Harms	#22	Plans for collecting, assessing, reporting, and managing solicited and spontaneously reported adverse events and other unintended effects of trial interventions or trial conduct	12
Auditing	#23	Frequency and procedures for auditing trial conduct, if any, and whether the process will be independent from investigators and the sponsor	12
**Ethics and dissemination**			
Research ethics approval	#24	Plans for seeking research ethics committee / institutional review board (REC / IRB) approval	16 - 17
Protocol amendments	#25	Plans for communicating important protocol modifications (eg, changes to eligibility criteria, outcomes, analyses) to relevant parties (eg, investigators, REC / IRBs, trial participants, trial registries, journals, regulators)	16 - 17
Consent or assent	#26a	Who will obtain informed consent or assent from potential trial participants or authorised surrogates, and how (see Item 32)	11
Consent or assent: ancillary studies	#26b	Additional consent provisions for collection and use of participant data and biological specimens in ancillary studies, if applicable	11
Confidentiality	#27	How personal information about potential and enrolled participants will be collected, shared, and maintained in order to protect confidentiality before, during, and after the trial	13

For peer review only

Declaration of interests	#28	Financial and other competing interests for principal investigators for the overall trial and each study site	23
Data access	#29	Statement of who will have access to the final trial dataset, and disclosure of contractual agreements that limit such access for investigators	13, 23
Ancillary and post trial care	#30	Provisions, if any, for ancillary and post-trial care, and for compensation to those who suffer harm from trial participation	NA
Dissemination policy: trial results	#31a	Plans for investigators and sponsor to communicate trial results to participants, healthcare professionals, the public, and other relevant groups (eg, via publication, reporting in results databases, or other data sharing arrangements), including any publication restrictions	16 - 17
Dissemination policy: authorship	#31b	Authorship eligibility guidelines and any intended use of professional writers	NA
Dissemination policy: reproducible research	#31c	Plans, if any, for granting public access to the full protocol, participant-level dataset, and statistical code	NA
**Appendices**			
Informed consent materials	#32	Model consent form and other related documentation given to participants and authorised surrogates	NA
Biological specimens	#33	Plans for collection, laboratory evaluation, and storage of biological specimens for genetic or molecular analysis in the current trial and for future use in ancillary studies, if applicable	NA

The SPIRIT Explanation and Elaboration paper is distributed under the terms of the Creative Commons Attribution License CC-BY-NC. This checklist was completed on 25. April 2023 using https://www.goodreports.org/, a tool made by the EQUATOR Network in collaboration with Penelope.ai

For peer review only
