# Supplementary material for: WAFER trial: a study protocol for a feasibility randomised controlled trial comparing wide-awake local anaesthesia no tourniquet (WALANT) to general and regional anaesthesia with tourniquet for flexor tendon repair
Source: BMJ Open. 2023 Aug 28;13(8):e075440. doi: 10.1136/bmjopen-2023-075440 (PMC10462963; doi:10.1136/bmjopen-2023-075440)
Supplement: Reviewer comments [file bmjopen-2023-075440.reviewer_comments.pdf]

PEER REVIEW HISTORY

BMJ Open publishes all reviews undertaken for accepted manuscripts. Reviewers are asked to complete a checklist review form (**http://bmjopen.bmj.com/site/about/resources/checklist.pdf**) and are provided with free text boxes to elaborate on their assessment. These free text comments are reproduced below.

ARTICLE DETAILS

**TITLE (PROVISIONAL)**	WAFER Trial: A study protocol for a feasibility randomised controlled trial comparing Wide-awake Local Anaesthesia No Tourniquet (WALANT) to General and Regional Anaesthesia with tourniquet for Flexor Tendon Repair
**AUTHORS**	Kanapathy, Muholan; Faderani, Ryan; Bray, Juliette; Dehbi, Hakim-Moulay; Panca, Monica; Vindrola-Padros, Cecilia; Prasad, Anjana; Burr, Nicola; Williams, Norman; Al-Ajam, Yazan; Bhat, Waseem; Wong, Jason; Mosahebi, Afshin; Nikkhah, Dariush

This article was not externally reviewed at BMJ Open. Protocols that have been independently assessed prior to submission to BMJ Open are usually fast-tracked to publication on the grounds that further substantial changes will not be possible. This independent assessment will usually be external, independent review for both a substantial grant award from a non-commercial or government funder and ethics approval.
